# Electrical facies of the Asmari Formation in the Mansouri oilfield, an application of multi-resolution graph-based and artificial neural network clustering methods

**DOI:** 10.1038/s41598-024-55955-0

**Published:** 2024-03-02

**Authors:** Seyedeh Hajar Eftekhari, Mahmoud Memariani, Zahra Maleki, Mohsen Aleali, Pooria Kianoush

**Affiliations:** 1 Department of Earth Sciences, Science and Research Branch, Islamic Azad University, 1477893855 Tehran, Iran; 2 Department of Petroleum and Mining Engineering, South Tehran Branch, Islamic Azad University, Tehran, Iran; 3 National Iranian Oil Company, Exploration Directorate (NIOC-EXP), Tehran, Iran

**Keywords:** Asmari reservoir, Electrofacies, Lithofacies, Zoning, MRGC and ANN clustering, Sandstone reserve, Sedimentology, Solid Earth sciences, Geology, Stratigraphy

## Abstract

Electrofacies analysis conducted the distribution effects throughout the reservoir despite the difficulty of characterizing stratigraphic relationships. Clustering methods quantitatively define the reservoir zone from non-reservoir considering electrofacies. Asmari Formation is the most significant reservoir of the Mansouri oilfield in SW Iran, generally composed of carbonate and sandstone layers. The stratigraphical study is determined by employing 250 core samples from one exploratory well in the studied field. Five zones with the best reservoir quality in zones 3 and 5 containing sandstone/shale are determined. Moreover, multi-resolution graph-based and artificial neural network clustering involving six logs are employed. Utilizing Geolog software, an optimal model with eight clusters with better rock separation is obtained. Eventually, five electrofacies with different lithological compositions and reservoir conditions are identified and based on lithofacies describing thin sections, sandstone, and shale in zones 3 and 5 show high reservoir quality. According to the depth related to these zones, most of the facies that exist in these depths include sandstone and dolomite facies, and this is affected by the two factors of the primary sedimentary texture and the effect of the diagenesis process on them. Results can compared to the clustering zone determination in other nearby sandstone reservoirs without cores.

## Introduction

The broad meaning of "facies" is the characteristics, appearance, and aspects of a rock unit, usually imaging the circumstances of its origin, particularly determining the unit from neighboring or associated units^1–4^. Electrofacies are numerical mixtures of petrophysical log reactions that reflect a rock interval's compositional characteristics and specific physicals. They are confined by multivariate techniques that contain discriminant, cluster, and Principal Components Analysis (PCA)^5–8^. Electrofacies specify reservoir rock properties, especially permeability, to simulate fluid flow in porous media. These are determined based on the classification of similar logs among different groups of logging data. Data classification is accomplished by statistical analyses such as principal component analysis, cluster analysis, and differential analysis^3,9–11^. A model of the reservoir's geological structure is also provided to create a three-dimensional model of a reservoir's properties, in addition to estimating porosity, permeability, water saturation, and other petrophysical properties. For geological modeling, lithofacies describe rock or sedimentary units with texture, structure, mineralogy, and rock properties, and these units can be employed to match important reservoir characteristics such as permeability and porosity^12–14^.

Therefore, identifying different rock facies of reservoir rock is a fundamental task in describing the characteristics of oil reservoirs. Detection of lithofacies in the repository is complex because the type and distribution of the facies are determined by the deposition system and are affected or altered by diagenesis and tectonics^1,4,15^. The most common technique for determining lithofacies is coring. The core data directly observes the lithofacies and can accurately distinguish the different facies. However, despite all the positive features, due to the high cost of core-extraction operations and the lack of 100% recovery, this method can only be employed in a small section of the field around the wellbore^1,14,16–18^.

On the other hand, core description is time-consuming and depends on the geologist's experience. Therefore, solving this problem requires a procedure cheaper than coring and capable of providing precision and separation of the rock facies to a suitable size similar to that of cores without wells^19–24^. An excellent way to respond to this demand is utilizing well-log data. Data loggers obtain indirect information from underground data and are much cheaper than kernels. Well-log calculations can be categorized into facies or electrical facies. Log facies can display rock and reservoir fluid properties and allow users to separate sedimentary units and layers. Created facies can be later applied to predict rock facies in intervals without core^11,25,26^. A practical method for facies analysis is to create a facies classification model that splits the log data into a set of log responses that describes sediment and provides sediment separation and identification from other sediments. This set of responses is called a cluster. These clusters have significant problems with dependency on the dimensionality, which differed from the geological distance and the two similar points in interpreting the cluster. They may not be geologically similar. The reason for this problem is the different views of geologists and loggers. This problem creates a nonlinear solid relationship between log and lithology and causes the problem of determining any partitioning of the log data into the associated sedimentary unit^17,22,27,28^.

The easy determination and application of this zoning and its reliance on log data make it possible to quickly and accurately distinguish reservoir segments from non-reservoir segments. With overgrowing the world, the cluster analysis method is utilized as the principal direction analysis of the log facies identification^4,7,29–31^. It is employed as an essential tool for logging in advanced petroleum software. Since these facies are determined only by pure mathematical processes in the cluster analysis method and no training or fitting function is considered, they are very accurate, and their application is not limited to a particular well or field. After identifying the log facies by assigning geological features to them, it can be applied to other wells on the surface of adjacent fields to match and predict the desired features^2,7,15,32,33^.

An essential advantage of electrofacies over alternative types of facies classifications of rocks in the subsurface is that electrofacies can be defined solely based on well-log responses without reliance on cores, cuttings, or outcrops. Although electrofacies are empirical, they are also objective; no subjective interpretations of sediment genesis or inferences about depositional environments are required. Moreover, there is no specific procedure for defining electrofacies. The general requirements could be defined from a uniform set of petrophysical log measurements with similarities between down-hole intervals are expressed quantitatively from the log responses. The intervals are consistently divided into subsets that have similar responses, and distinctions between subsets are expressed as mathematical functions^3,7,34^.

As the literature review of recent years, Serra and Abbott^35^, and Serra^36^ defined electric facies as "a set of responders that, in addition to characterizing the sediments, allows them to be separated." Wolf and Pelissier-Combescure^37^, and Selley^38^ introduced the first automated method for classifying "facsimiles" into electrical facies. Tavakkoli and Amini^39^ employed a set of logs instead of one log to assign more features to a particular logfacies simultaneously. In 2015, some studies of identifying and interpreting electrical facies employing the Self Organized Neural Network (SONN) method and its prediction for sedimentary facies in the Asmari reservoir of an SW oilfield of Iran have been carried out^26,40^. Kiaei et al.^26^ Modeled three-dimensional reservoir electro-physics using integrated clustering and geostatistical methods in the Persian Gulf. Their approach included Hierarchical Cluster Analysis (HCA), Multi Resolution Graph-based Clustering (MRGC), and self-Organized Maps (SOM). Based on their results, hierarchical clustering as a robust and practical approach to data clustering was performed based on profile validation and geological information.

El Sharawy and Gaafar^41^ zoned the reservoir based on statistical analyzes in the Nubian sandstone Gulf of Suez, Egypt. They showed that the entire reservoir could be divided into at least four electro physics with significant changes in reservoir quality, which correlated with permeability changes. Tian et al.^42^ employed the multi-resolution graph-based clustering (MRGC) method for determining the Electrofacies (EF) and Lithofacies (LF) from well-log data obtained from the intraplatform bank gas fields located in the Amu Darya Basin. Rastegarnia et al.^9^ predicted 3D Flow Zone Index (FZI) and electrified (EFACT) volumes from a large volume of 3D seismic data. Rafik and Kamel^16^ evaluated the permeability of the formation for a sandstone reservoir in the South Ramsey reservoir formations from log well data utilizing multivariate methods. Their results showed that permeability prediction using variable selection to non-parametric Alternating Conditional Expectations (ACE) regression is the best way to predict permeability. Kadkhodaie and Kadkhodaie^27^ reviewed different reservoir rock typing approaches from geology to seismic and dynamic and proposed an integrated rock type workflow for worldwide carbonate reservoirs. Jafarzadeh et al.^18^ investigated the distribution of reservoir facies employing the available data to identify areas that are prone to be considered in the production and development plans of the field in terms of their storage and fluid flow capacity. Eventually, the experimental results illustrated that the adaptive multi-resolution graph-based clustering algorithm for electrifying analysis also outperformed the original MRGC approach on clustering and propagation prediction with higher efficiency and stability^34,43,44^. Recently, the electrifies predicted using the MRGC approach to generate rock mechanical properties such as Young's modulus, Poisson's ratio, unconfirmed compressive strength, and internal friction coefficient^5,45,46^. Kianoush^47^ estimated an ANN based model of formation Pressure for the Azadegan hydrocarbon Reservoir, SW Iran. Finally, Okhovvat et al.^8^ used Kernel Principal Component Analysis (KPCA) to improve the performance of electrical facies classification.

This study addresses some of the ambiguities of using drilling cuttings and logs data in analyzing petroleum formations by combining the results of logs and describing log facies. It is mainly done by defining the log facies and the reservoir zoning of the study well. Moreover, as an innovation, the wells are zoned employing well-logging diagrams, and then, utilizing MRGC and ANN techniques, electrical facies from log data are determined. Also, as a geological novelty, a zoning methodology for the Asmari Formation in the Mansouri field is considered. Two general lithofacies of carbonate-evaporitic facies and siliceous-clastic petrofacies are determined, describing thin sections that have acceptable consistency and similarity with electrofacies clustering of sandstone in deeper parts of Asmari reservoir. Furthermore, integrating ANN and MRGC clustering methods, instead of the limited and unique information of a few cored wells, comprehensive information on the simulated sedimentary facies is obtained in all the field wells, which immensely helped the sedimentary modeling of the studied field.

## Geological setting

### Structural geology

**Figure 1:**
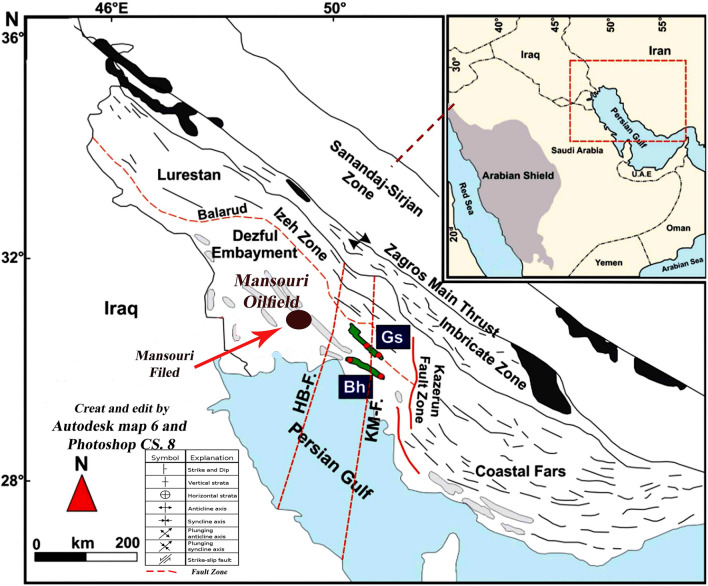
Location of Mansouri oilfield in the Dezful embayment, SW of Iran (create and edit by Autodesk Map 6)^6,21,50^.

Mansouri field in the southernmost part of the north Dezful zone, about 45 km south of Ahwaz, is located approximately on the border of the Arabian Plate, and quaternary alluviums represent the Zagros Plate and its surface outcrop. Mansouri field is located in the north of the Ahwaz field, in the west, in the vicinity of the Abteymur and Susangerd fields, and the northeast of the Shadegan field. The axial trend of this field is from the northwest to the southeast (the general Dezful embayment) and lies between 48° to 52° east longitude and 30° to 32° north latitude (Fig. 1)^6,21,40^. Mansouri field is located in a flat zone just off the foot of the foothills and was discovered by seismic exploration in 1963. Based on the seismic and structural maps of the Mansouri field, it is an anticline with gentle and low slopes in the northwest-southeast (NW–SE) direction. The northern slopes are slightly higher than the southern ones^7,24,40,48–50^.

Furthermore, 5–6°, the slope of the eastern and western slopes is about 1–5°. The study of geophysical maps and the information on drilled wells show no evidence of fault or disruption in the field, and it is generally mild in structure (Fig. 1). Mansouri's field in the horizons of Asmari is about 42 km long. It has a variable width of up to 6 km in the middle of the field and an average of 4.5 km, which decreases to the east and west slopes. The dimensions of the reservoir at the contact surface of water and oil (2272 m below sea level) are 30 km long and 3.5 km wide, stretching northwest-southeast^4,32,51–53^.

The limestone Asmari Formation is the most critical Zagros sedimentary reservoir. In terms of sedimentation, it began in the former Oligocene and continued until the former Miocene. This formation is the shallow horizon of oil producers in SW Iran and forms the most essential rock reservoir in Dezful's embayment. The formation's upper and lower limit has the same slope. In addition to the Asmari reservoir and sandstone section of Ahwaz, the Bangestan reservoir (Ilam and Sarvak Formations) are also present in this field^39,50^.

### General stratigraphy

**Figure 2:**
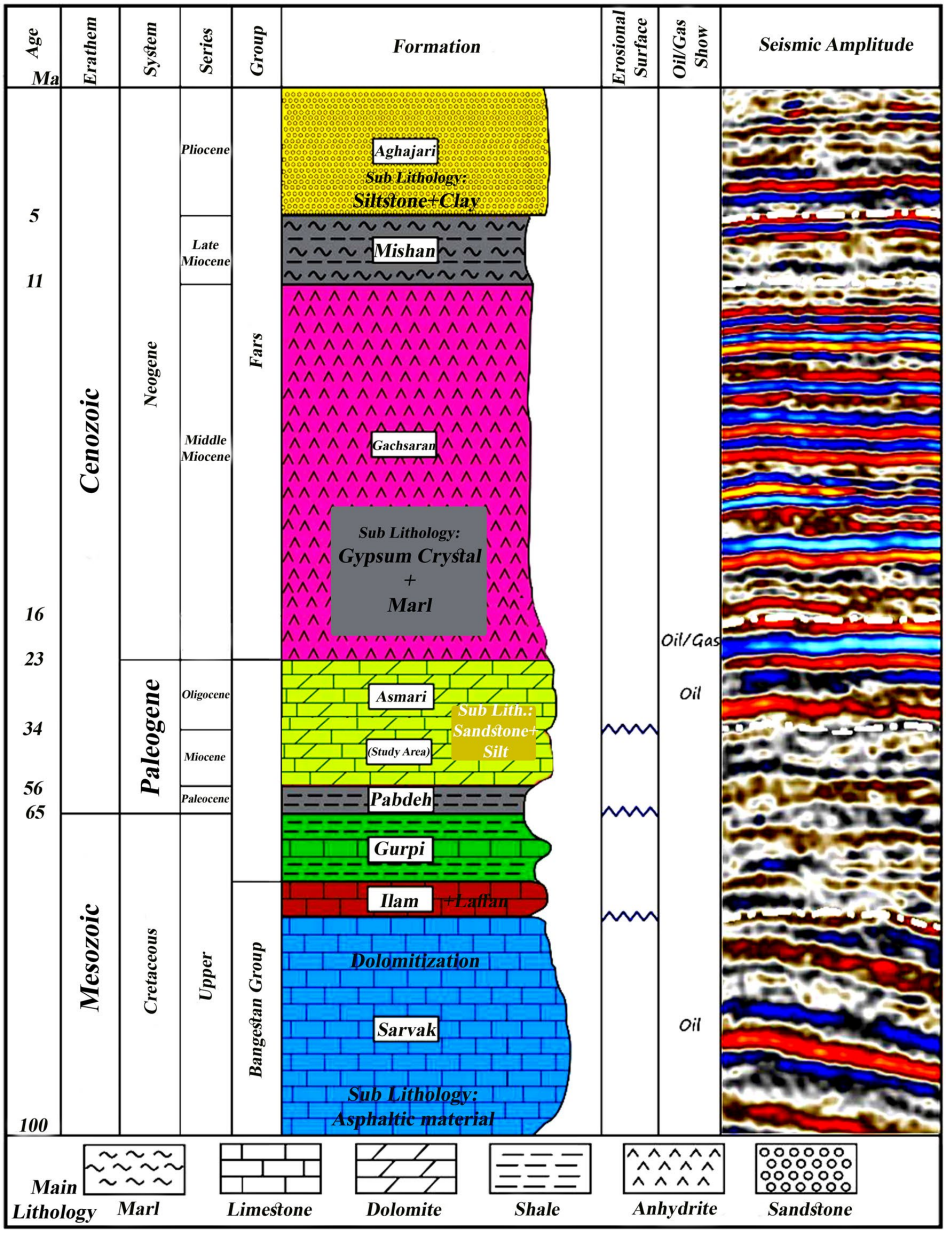
General stratigraphic column of Masouri oilfield with depicting study area^12^.

The stratigraphic column, reservoir zonation, and selected petrographic thin sections of the Bangestan reservoir are presented in Fig. 2 for one of the drilled wells. The Bangestan reservoir was subdivided into nine zones in the studied field based on petrography, petrophysical parameters, and well-logging data^10,12,20,54^.

The Asmari Formation geologically consists of sandstone, dolomite, limestone and low to average clay minerals. This formation is characterized by light brown, hard and fine-grained dolomite with lime layers, followed by medium to coarse grains, often without cement or calcareous and dolomite and sandy cement. White limestone to light grey is observed in the formation's lower part.

In Ilam Formation, the dominant lithology is White wackestone–mudstone, Brown packstone–wackestone, and White to brown limestone and argillaceous in some layers.

In Sarvak Formation, the dominant lithology is brown wackestone–packstone with dolomitization in upper intervals and dense limestone with asphaltic material in lower intervals.

In the bottom of the last zone, a gradational variation from the Kazhdumi shale to Sarvak limestone indicates a regressive environmental condition. The porosity data variation indicated that the reservoir quality would improve from the base to the top^12,28,52,55^.

## Methodology

Electrofacies (electrical facies) are identified using petrophysical logs such as gamma ray, resistivity, acoustic, density, and neutron. Since logs measure rocks' physical characteristics, electrofacies can be attributed to one or more lithofacies. Determination of facies is one of the main components of oil exploration and determination of reservoir properties^28,30^.

### Estimating the number of distinct electrofacies

Due to electrofacies being determined experimentally, the number of different electrofacies is relatively incidental. The number of proper electrofacies is partially conditional to the number of log properties employed in their calculation and the joint nature of the statistical distributions of the log measurements. It also reflects the intention of electrofacies classification and the technique for assessing and employing the final classification. A simple recognition between reservoir and non-reservoir rock may be completed with an electrofacies classification of only two classes. At the same time, an investigation for environmental interpretation may demand a dozen or more classes^5–7^.

Due to a restricted number of well-logs that measure different physical effects in the employed well here, a proper electrofacies interpretation will only involve a few facies classes. Determining the proper number needs trial and error, beginning with many classes and lessening the number to eliminate trivial categories that contain only a few rare observations or combining ill-defined classes with comparable properties. The exact trial-and-error process can be utilized to assess alternative approaches like additional clustering algorithms^3,42^.

The pursuit of cluster analysis is to divide the data set into specific groups based on the similarity or differences between the groups. The data in each group have the most similarity and differences from those in other groups. The usual method for identifying and separating different sedimentary facies is to study thin sections prepared from core samples. However, the problem is the need for sufficient information on cores in the reservoir area. This information is available only in a very limited number of cored wells. One geological concern is preparing and explaining the sedimentary model with this limited information^5,13–15,49^. This study created an electric facies model utilizing a clustering method and well logs in a drilled well with coring in the Asmari Formation. Then it was compared with different clustering methods to get the best model, and then it was adjusted and improved with the facies obtained from the cored well logs and finally implemented in all the wells. In this regard, instead of the limited and unique information of a few cored wells, complete information on the simulated sedimentary facies was obtained in all the field wells, which significantly helped the sedimentary modeling of the studied field. In this research, MRGC and ANN clustering methods are used to determine electrical facies, which will be discussed in the following:

### MRGC clustering method

**Figure 3:**
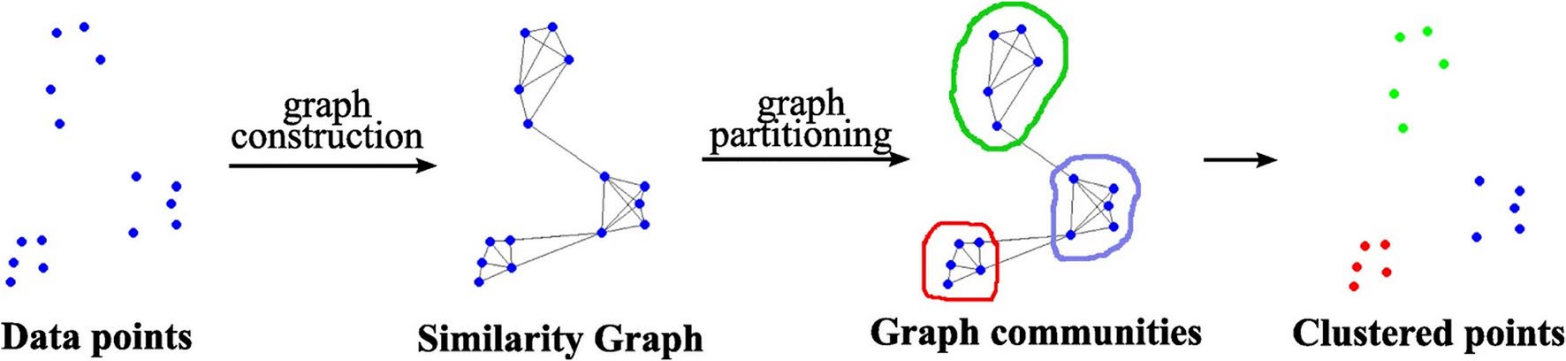
Multi graph-based data clustering via multiscale community detection^5,6^.

MRGC is one of the few non-parametric and very suitable methods for studying and analyzing data clusters obtained from well logs and drilling cores. This method has several benefits, such as the capability to identify natural patterns in the logs, no need for prior knowledge about the data, an automatic suggestion of the best number of clusters, the lowest parameters and insensitivity to their changes, and no restrictions on the type and number of data and clusters^5,12,34,42^. Schematic diagram of the workflow for multi graph-based clustering is presented in Fig. 3.

The data underlying structure is examined, and natural data groups that could have dissimilar shapes, sizes, densities, and relative separations are formed. MRGC defines the optimal number of clusters automatically, and the level of EF clusters can be specified based on actual requirements. It has a stable outcome and can efficiently partition the EF and indicate the LF from the well-log data. This approach involves the following two parameters to make the MRGC method more robust than other hierarchical clustering algorithms. Neighboring Index (NI) is a parameter based on the weighted rank of measurement point *x *relative to all other measurement points *y*. Two points close to each other can be easily separated if they have a high *NI*(*x*). Subsequently, the number of facies can be easily specified using the Eqs. (1) to (4)^6,18,34,42^.

1 $$s(x) = \sum\limits_{n = 1}^{N = 1} {\exp ( - m/\alpha )}$$

2 $$s_{\min } = \mathop {\min }\limits_{i = 1,N} \left\{ {s(x_{i} )} \right\}$$

3 $$s_{\max } = \mathop {\max }\limits_{i = 1,N} \left\{ {s(x_{i} )} \right\}$$

4 $$NI(x) = \frac{{s(x) - s_{\min } }}{{s_{\max } - s_{\min } }}$$

where *N *is the total number of data points, *x *is the m_th_ nearest neighbor of *y *(*m* ≤ *N* − 1), and α is the smoothing parameter greater than zero. *NI*(*x*) varies from 0 to 1, and as the value of *NI*(*x*) increases, the point becomes closer to the kernel of a cluster.

Kernel representative index (KRI) is a parameter that combines *NI*(*x*), the neighbor function *M*(*x*, *y*), and the distance function *D*(*x*, *y*). The factor *NI*(*x*) allows us to recognize the kernel of a cluster. The number of neighbors *M*(*x*, *y*) tends to generate clusters of equivalent size and clusters of equivalent volume from the distance *D*(*x*, *y*). Combining these two factors produces a sufficient balance between a cluster's size and volume and generates consistent outcomes. KRI is estimated by employing the Eq. (5)^1,6,34,42^.

5 $$KRI(x) = NI(x).M(x,y).D(x,y)$$

where *M*(*x*, *y*) = *m*, when *y *is the m_th_ neighbor of *x*, and *D*(*x*, *y*) is the *x *and *y distance*.

First, the kernel points that influence all their neighboring data points (call members) are specified, and subsequently, all members are compared. The members affected by the kernel point affected the other members as well. Boundaries are assigned when a member is affected by its previous member but without affecting others. The NI of each point in the data set is calculated to identify the clusters. Subsequently, small natural groups of the points are formed based on the NI to determine a KNN attraction for each point. Independently, an optimal number of clusters is computed based on the KRI. Eventually, the terminal clusters are constructed by merging the small clusters^1,5,6,9,18,26,34,42^.

To determine the electrofacies with this method, first in the FACIMAGE™ section of the Geolog software, among the petrophysical logs, those logs that are most related to the results target, which include: gamma ray log (CGR), sonic log (DT), density log (RHOB), neutron log (NPHI) and water saturation (SW), is selected.

Data clustering is the basis of modeling and classification algorithms. This process aims to define small natural and essential groups from a large data group^27,40^. The MRGC method uses two indices NI (neighborhood index parameter) which determines the proximity of each point in a data set to the peak or trough of the probability density function of the data, and KRI (Kernel Representative Index), is an index used to determine points prone to represent the core or center of the cluster.

Define the neighborhood characteristic of any graph G without isolated vertices to be Eq. (6):

6 $$n.Char(G) = s_{1} - s_{2} + s_{3} - ....,$$

where: s_i_ is the number of subsets of V (G) of cardinality i that are externally dominated, meaning that ${\text{S}} \subseteq {\text{N(}}\nu {)}$ for some $\nu \in {\text{V(G) - S}}$. Thus *s*_1_ = *n* is just the order of *G*, and *s*_2_ is the number of pairs of vertices that have a common neighbor.

The neighborhood characteristic of a graph can also be calculated in terms of the complete bipartite subgraphs present in G. For every graph G without isolated vertices Eq. (7):

7 $$n.Char(G) = 2\sum\limits_{1 \le i \le j}^{{}} {( - 1)}^{i + j} k_{i,j}$$

where: $k_{i,j}$ count the number of complete bipartite^5,56^.

Kernel functions allow mapping non-linearly separable points into a (generally) higher dimensional feature space so that the inner product in the new space can be computed without needing to compute the exact feature maps bringing further computational benefits. Although most algorithms in the broader field of machine learning have been developed for the linear case, real-world data often requires nonlinear models capable of unveiling the underlying complex relationships towards improving the performance of downstream tasks^5,8,34,57^. To use kernel functions for learning node representations via matrix factorization:

Let (X, d_X_) be a metric space and H be a Hilbert space of real-valued functions defined on X. A Hilbert space is called reproducing kernel Hilbert space (RKHS) if the point evaluation map over H is a continuous linear functional. Furthermore, a feature map is defined as a function $\Phi :X \to {\rm H}$ from the input space X into feature space H. Every feature map defines a kernel $K:X \times X \to R$ as follows (Eq. (8))^8,57^:

8 $$\begin{aligned} & K(x,y) = \left( {\Phi (x),\Phi (y)} \right) \hfill \\ & \forall (x,y) \in X^{2} \hfill \\ \end{aligned}$$

It can be seen that $K(x,y)$ is symmetric and positive definite due to the properties of an inner product space^5,8,57^.

### Artificial neural networks (ANN) clustering method

**Figure 4:**
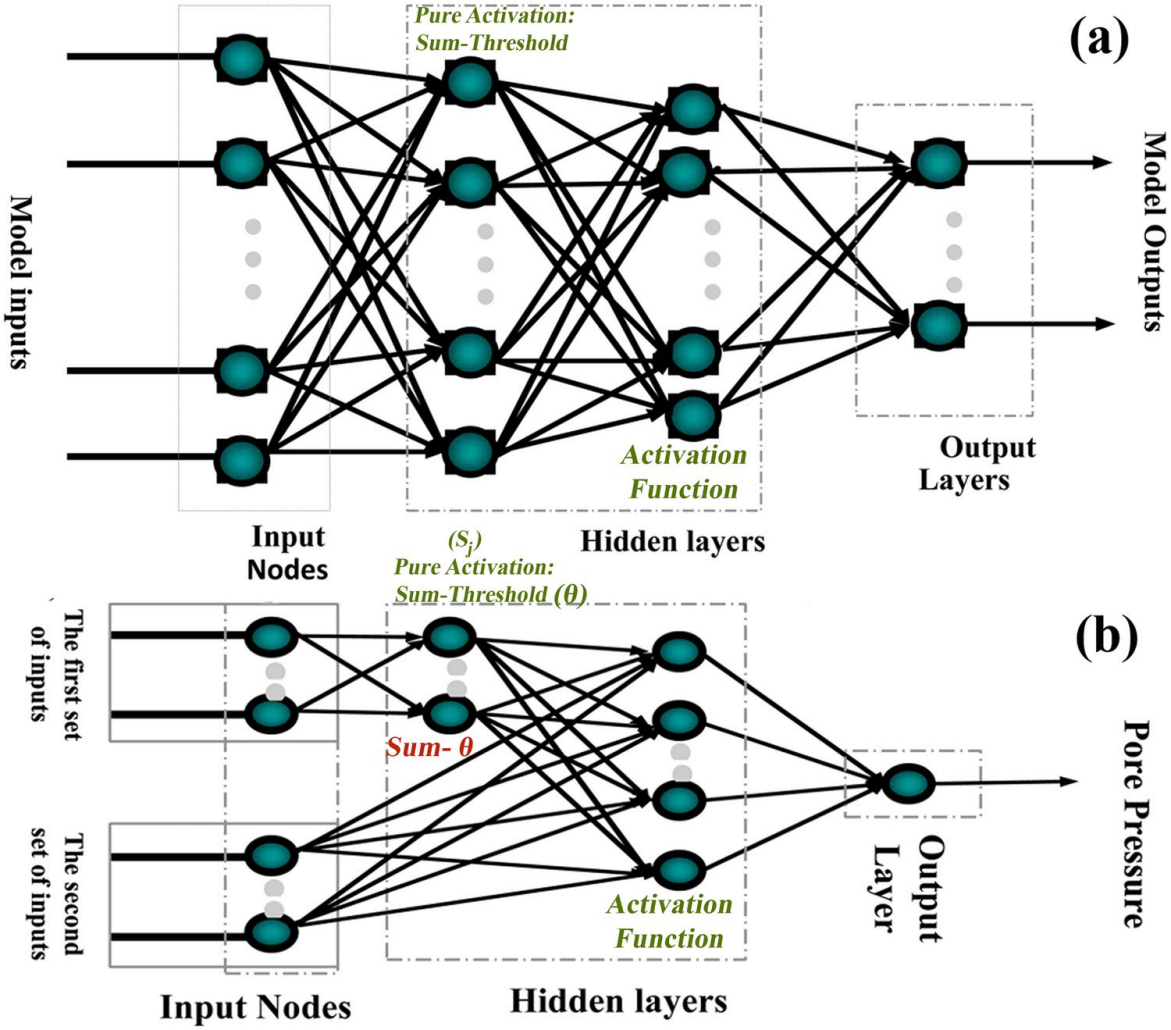
(**a**) Structure or topology of a typical forward neural network, (**b**) FFBPANN structure made by “Lianbo Hu” to predict pore pressure^43,59^.

A new method for determining electrical facies is applying artificial neural networks. The method teaches the network to produce a specific output based on the input data. Here, the network is trained using different algorithms. The network's training uses inputs whose output is also applied to the program. The trained network can now produce the desired output for new inputs. Each neural network has at least three layers (Fig. 4a). These layers include input, output, and intermediate (hidden) layers. Each input has its weight and enters the hidden layer using the combination function. The simplest mode of the combination layer is multiplying each input by weight and summing these numbers. This number is transferred to the output layer using the transfer function in the hidden layer. Some percent of the data are also used for comparison^7,9,44,47,58,59^.

The network node or artificial neuron acts as the biological neuron, and the connection weight of the neural network functions as the chemical transmitter and the electrical transmitter. ANN can intelligently analyze with simple mathematical methods and deal with nonlinear, fuzzy, and complex relationships. A neuron is the main element of an ANN, the model. The function of artificial neurons, as the name implies, is to simulate biological neurons. The artificial neuron has a P input and an output^44,59^.

The inputs are x_i_ (i = 1 , …, p), and the output is y_j_. The relationship between inputs and outputs can be set as follows (Eq. (9)):

9 $$\left\{ {\begin{array}{*{20}l} {S_{j} = \mathop \sum \nolimits_{i = 1}^{p} w_{ij} .x_{i} - \theta_{j} } \\ {y_{j} = f\left( {S_{j} } \right)} \\ \end{array} } \right.$$

Here $\theta$ is the threshold. W_ij_ is the weight or weight of the connection from signal i to neuron j. S_j_ is pure activation, and f (S_j_) is the activation function^43,58^. There are many activation functions, including linear, ramp, threshold, crushing, etc. Neurons are arranged in different ways depending on the type of network.

There are several types of connections or weighted links in neural networks:

- Forward: Most links are of this type in which the signals move in only one direction. There is no feedback (loop) from input to output. The output of each layer does not affect the same layer.
- Backward: Data is fed back from the nodes of the upper layer to the nodes of the lower layer.
- Lateral: The output of the nodes of each layer is used as the input of the nodes of the same layer.

The Feed-Forward Back Propagation Artificial Neural Network (FFBPANN) is widely used in petroleum engineering applications. The structure or topology of the feed-forward neural network is shown in Fig. 4b^32,34,58,59^.

The number of neurons significantly affects the prediction results; in the studied well, the Feed Forward-Backpropagation (FFBP-ANN) model based on the Lianbo Hu ANN method, the best combination of neurons is 2-5-1 format. It means that the first layer has two neurons, the second layer has five neurons, and the output layer has one neuron. Supplementary data are presented as an appendix at the end of the manuscript.

## Results

**Figure 5:**
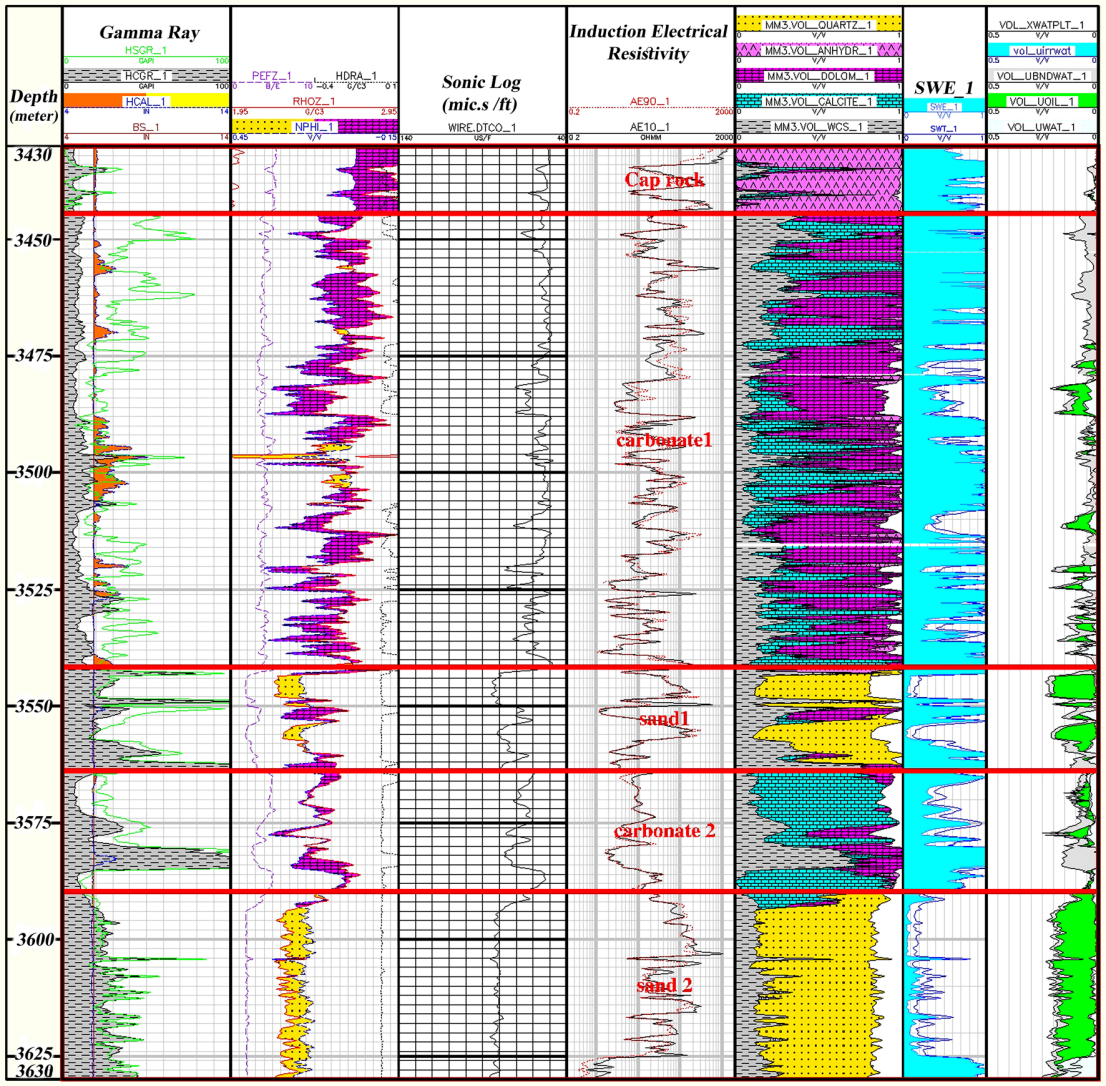
Reservoir zonation sequences of the Asmari Formation based on lithological alteration in the studied well.

In the sequence of oil/gas wells, zoning is one operation that segregates the sequence studied into zones with common conditions (geological or reservoir conditions, etc.). This section used log data to represent the Asmari Formation in the Mansouri Field accurately. Zones with typical reservoir geology (lithology) are studied in the well sequences using read diagrams. Figure 5 shows the sequence of the study well after zoning.

After evaluating the sequence of the Asmari Formation, the petrophysical parameters are averaged to determine the best production intervals in terms of oil accumulation in the well, and the best intervals with the highest hydrocarbon volume are determined. The net production zone is an essential indicator in evaluating reservoir quality. This thickness comprises a part of the reservoir with acceptable reservoir and petrophysical conditions. Shear limits are determined to achieve this goal for some of the calculated petrophysical parameters. These intervals are the zones where the focus of the reservoir studies is concentrated. Based on zonation in wells, the cost and technology for cutting boundaries differed.

The shear boundaries for the carbonate and sandstone sequences of the Asmari Formation are presented in Table 1.

**Table 1:** Cutting off limits for carbonate and sandstone sections employing shale volume, water saturation, porosity and oil volume.

Parameter	Type	Cut off (%) Carbonate	Cut off (%) Sandstone
PHIE	≥	4.5	8
SWE	≤	50	50
Vsh	≤	20	30

The cut of sandstone and carbonate values are determined from prepared well logs and calculating shale volume, saturation of water, porosity percent, and oil volume. More details of the calculations for each zone are presented in Table 2.

**Table 2:** Average petrophysical parameters calculated per weight for different zones in the Asmari Formation.

Interval (m)	Zone	Vsh (%)	PHIE (%)	SWE (%)	Uoil (%)
3427.5 to 3444.5	Zone-1	2.7	2.4	99	0.01
3444.5 to 3560.5	Zone-2	18.1	3.9	91.8	0.055
3560.5 to 3570.5	Zone-3	27.4	13.1	46.5	7.4
3570.5 to 3585	Zone-4	11.5	8.9	76.7	2.9
3585 to 3630	Zone-5	29.5	12.9	63	6.3

### Stratigraphy of the studied field

In the data preparation stage, after loading the data into the software, their quality in terms of readings, well control conditions, and logs used is checked. Afterward, the pre-computational stage is performed using well information, which finally obtained information about the well conditions and drilling mud properties in the evaluated sequence. Then environmental correction of the data is performed, and the effects of wells, drilling mud, etc., are removed from the logs readings, and consequently, zoning is performed using charts. In the next step, the lithology is evaluated and estimated in each sequence using corrected and edited logs and lithology cross-sections (neutron-density, Rho-U plot, MID plot, and MN plot). Finally, employing the probabilistic method, the petrophysical parameters are calculated in the whole sequence, and the average of these parameters is calculated in the whole well and each zone. The production zones are also identified in the wells studied using the average petrophysical parameters and determining the cutoff for some of these parameters.

In the sequence of oil/gas wells, zoning is one procedure that segregates the sequence studied into zones with common conditions (geological or reservoir conditions, etc.). This section used log data to represent the Asmari Formation in the Mansouri Field accurately. Zones with typical reservoir geology (lithology) are studied in the well sequences using read diagrams.

Based on petrophysical results, the Asmari Formation has been divided into five zones (Fig. 5).

**Figure 6:**
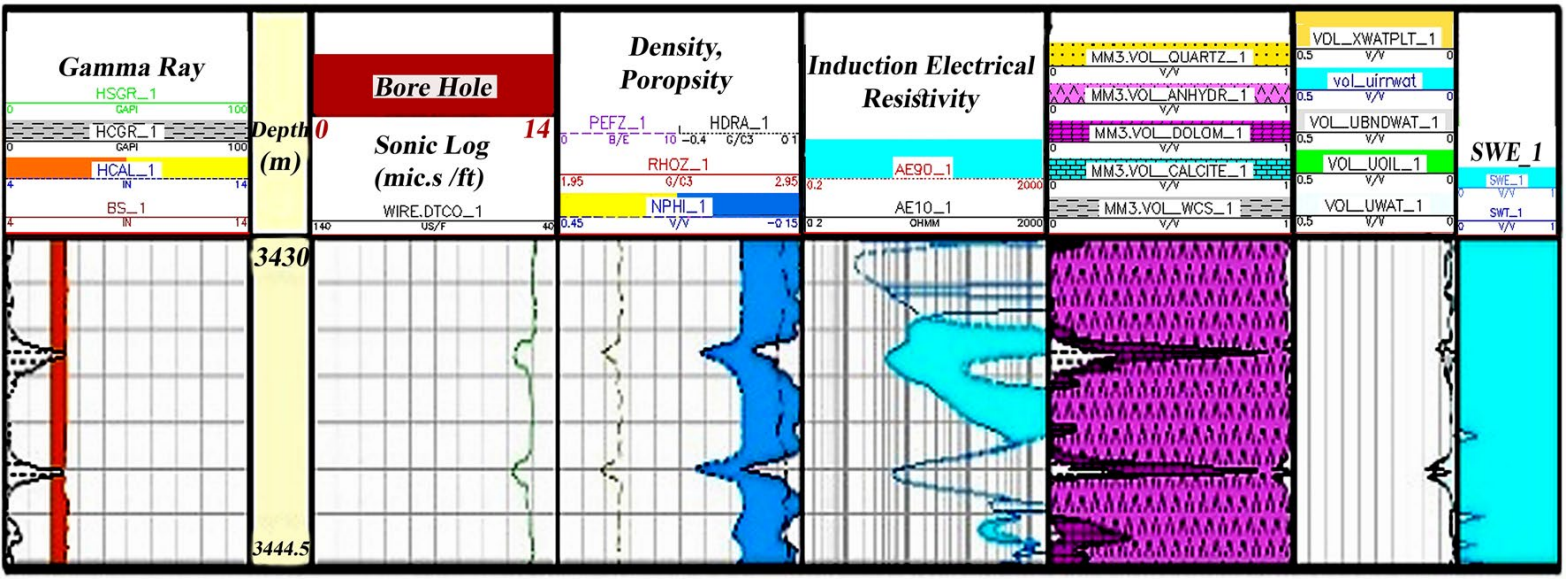
Initial petrophysical assessment based on lithology zoning segmentation 1.

Zone 1 (3444.5–3427.5 m): This zone exists in all drilled wells. The central part of the zone consists of dolomite plus a thick layer of limestone. Limestones are mostly cream to light brown and cream to gray, semi-hard to hard, fine-grained, micro-crystalline with anhydrite, chert, mudstone argillic to packstone (Fig. 6).

**Figure 7:**
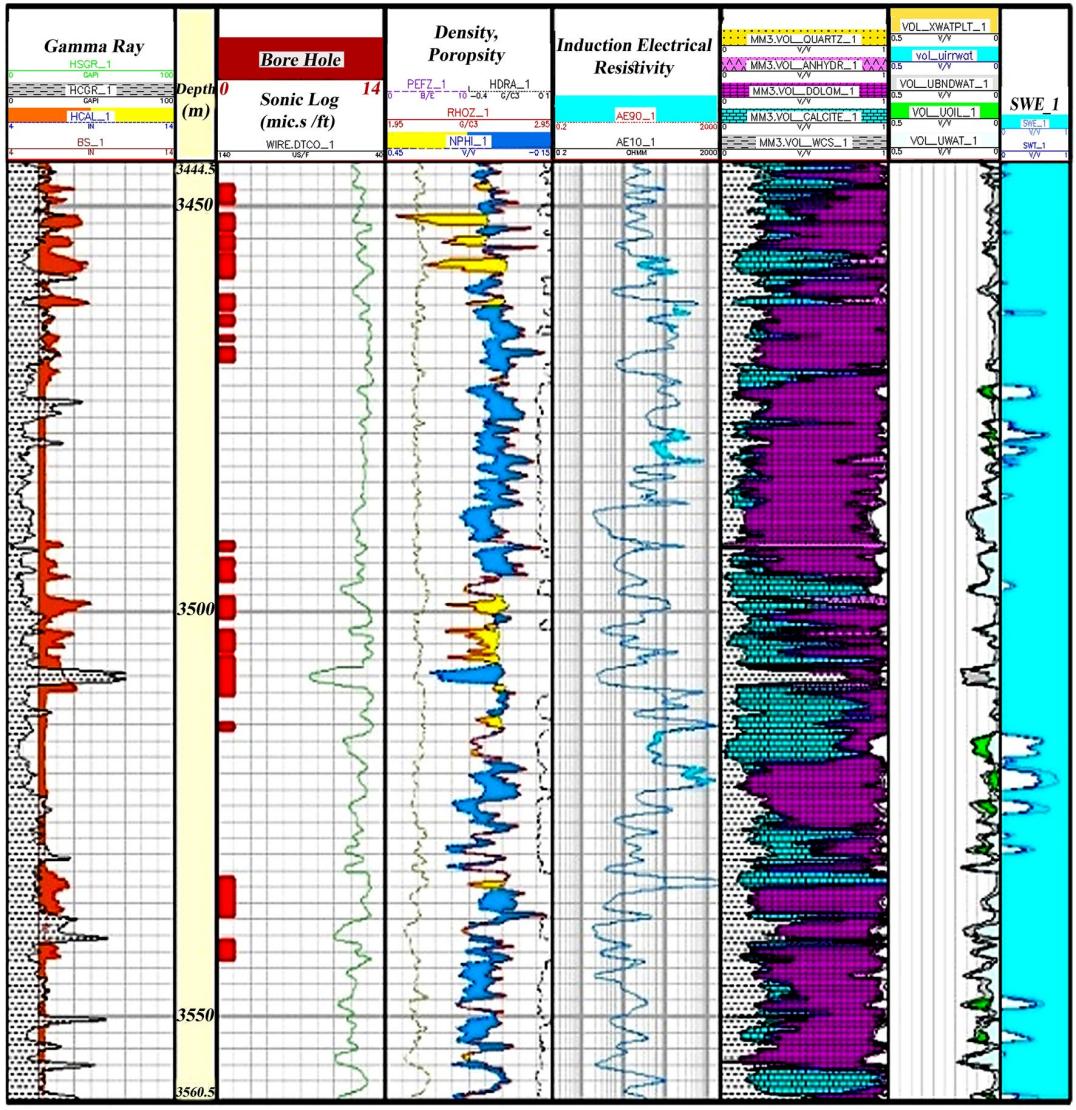
Initial petrophysical assessment based on lithology zoning segmentation 2.

Zone 2 (3560.5–3444.5 m): This zone is present in all wells. The lithology of this zone mainly consists of dolomite and sandstone (Fig. 7).

**Figure 8:**
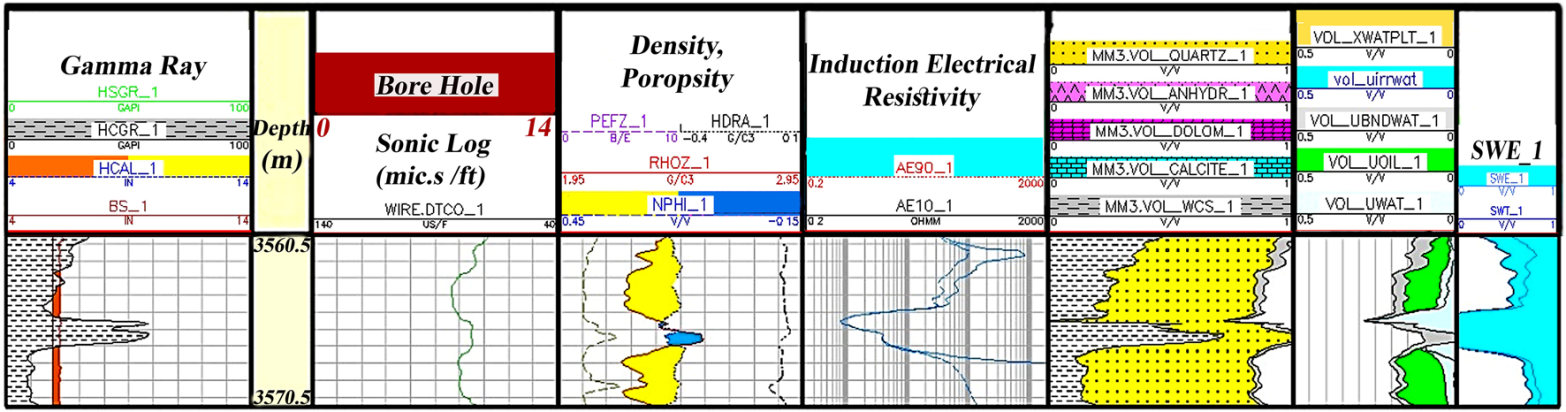
Initial petrophysical assessment based on lithology zoning segmentation 3.

Zone 3 (3570.5–3560.5 m): Its dominant lithology includes shale, limestone, and sandstone (Fig. 8).

**Figure 9:**
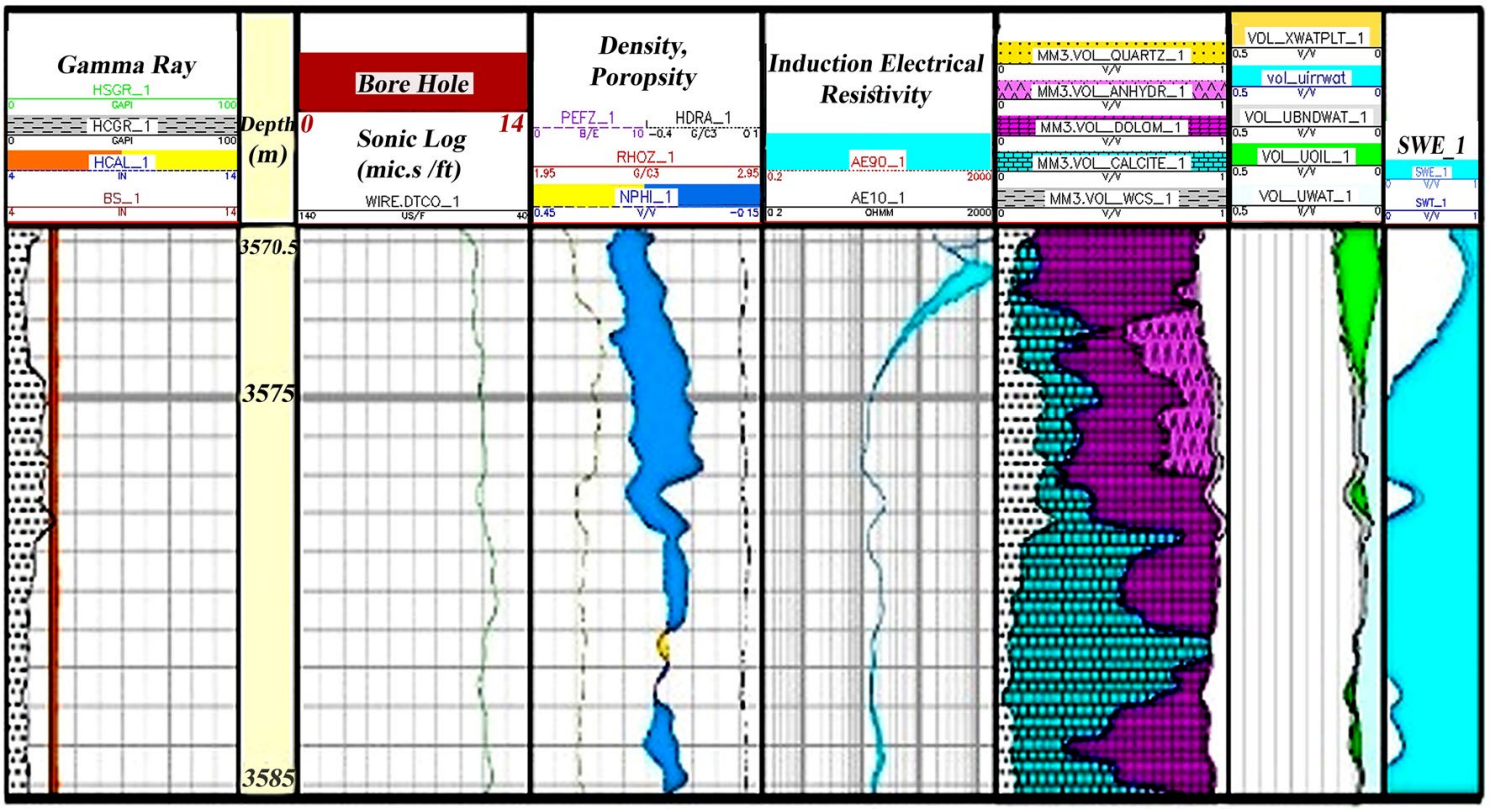
Initial petrophysical assessment based on lithology zoning segmentation 4.

Zone 4 (3585–3570.5 m): This zone exists in all wells, and most of it contains a barrier/ beach ridge and is likely to be associated with the Ahwaz sand dunes. Much of the lithology of this zone is sandstone and shale (Fig. 9).

**Figure 10:**
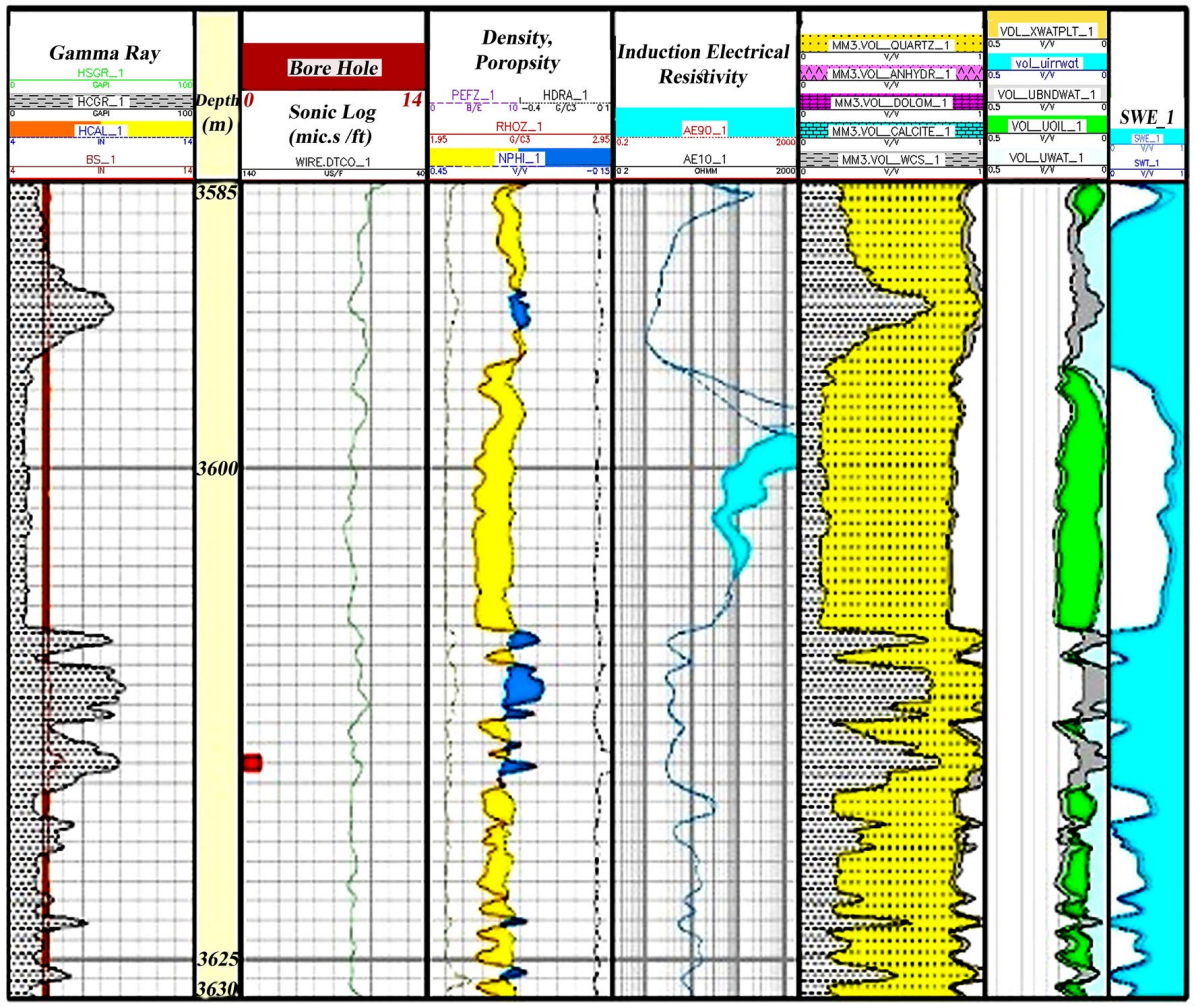
Initial petrophysical assessment based on lithology zoning segmentation 5.

Zone 5 (3630–3585 m): This zone cannot be identified in all wells due to a lack of logging data. The main lithologies in this zone are shale, sandstone, and limestone (Fig. 10).

### Zoning

According to stratigraphical and petrophysical results, the segmentation zone 1 is 14.3 m thick in the well sequence. The dominant lithology of this zone is mainly anhydrite. A small percentage of shale and dolomite is also found in this zone. Due to the petrophysical property of anhydrite, this zone plays no role in the Asmari Formation in the well-studied. It has very low porosity and almost no hydrocarbon accumulation in this zone (Fig. 6).

According to Fig. 7, second zone, which has the most sequence in the studied well, is 116 m thick and has the dominant lithology of calcite and dolomite. At some intervals, there are pores in the zone with little oil accumulation. The average porosity in this zone is also low and has high water saturation and, consequently, low oil volume.

The third segment in Fig. 8 has a minimum thickness of about 10 m from the study well. It has good oil accumulation capacity with sand and shale lithology. The high porosity, and low water saturation with good hydrocarbon volume, make this zone the main reservoir zones in the studied well sequenced.

The lithological conditions in of fourth segment and zone (Fig. 9) are approximately the same as those of the second zone, with the dominant lithology being calcite and dolomite containing shale and anhydrite. Its thickness is 15 m. This zone has little porosity, and the water saturation is somewhat high, resulting in small quantities of oil.

Fifth zone occupies the last part of the Asmari Formation sequence in the studied well (Fig. 10) and is the main reservoir zone in the studied sequence. The lithology of this zone is similar to that of the 3rd zone, sand and shale zones, which have high porosity, low water saturation, and consequently higher oil accumulations than other zones. The zone is 45 m thick.

The average calculated petrophysical parameters of studied zones employing well-A are shown in Table 2. According to the information presented in this table, it can be seen that the average petrophysical parameters in zones 3 and 5 have the best reservoir conditions compared to other zones. Due to the consequence of these two zones, the main reservoir zones in the Asmari Formation sequence are studied in shaley sandstone lithology.

As depicted in Table 2, the shale volumes in zones 3 and 5 are 27.4% and 29.4%, respectively. Furthermore, porosities are 13.1% and 12.9%, water saturations are 46.5% and 63%, and oil volumes are 7.4% and 6.3% respectively. These facts show the clear distinction between these two zones and other zones in terms of the reservoir capacity of sandstone and shale in them. The following will discuss clustering methods to study electrofacies and compare them with lithofacies.

### Determination of electrical facies

The facies are categorized using appropriate electrical log readings and proper clustering methods. The total sedimentary units with lithological features, sedimentary structure, geometrical form, fossil appendages, longitudinal flow patterns, and similar layering surfaces are distinguishable from the characteristics mentioned above by facies^38,60^. Well logs are one of the primary sources of subsurface information in oil/gas fields. These tools exhibit features such as mineralogical composition, texture, sedimentary structures, and reservoir properties (such as porosity and permeability) directly or indirectly^35,36^. Nowadays, determining electrical facies in reservoir formations is one of the standard studies in the characterization of reservoirs. The wide use of these facies and their flexibility to determine specific reservoir parameters due to the type of input data has today made this method one of the most potent tools in reservoir studies. Facies data are used in cases such as separating reservoir zones from non-reservoir and in field-scale and large-scale structural adaptation. This data's importance is called the virtual core^8,18,50^.

**Figure 11:**
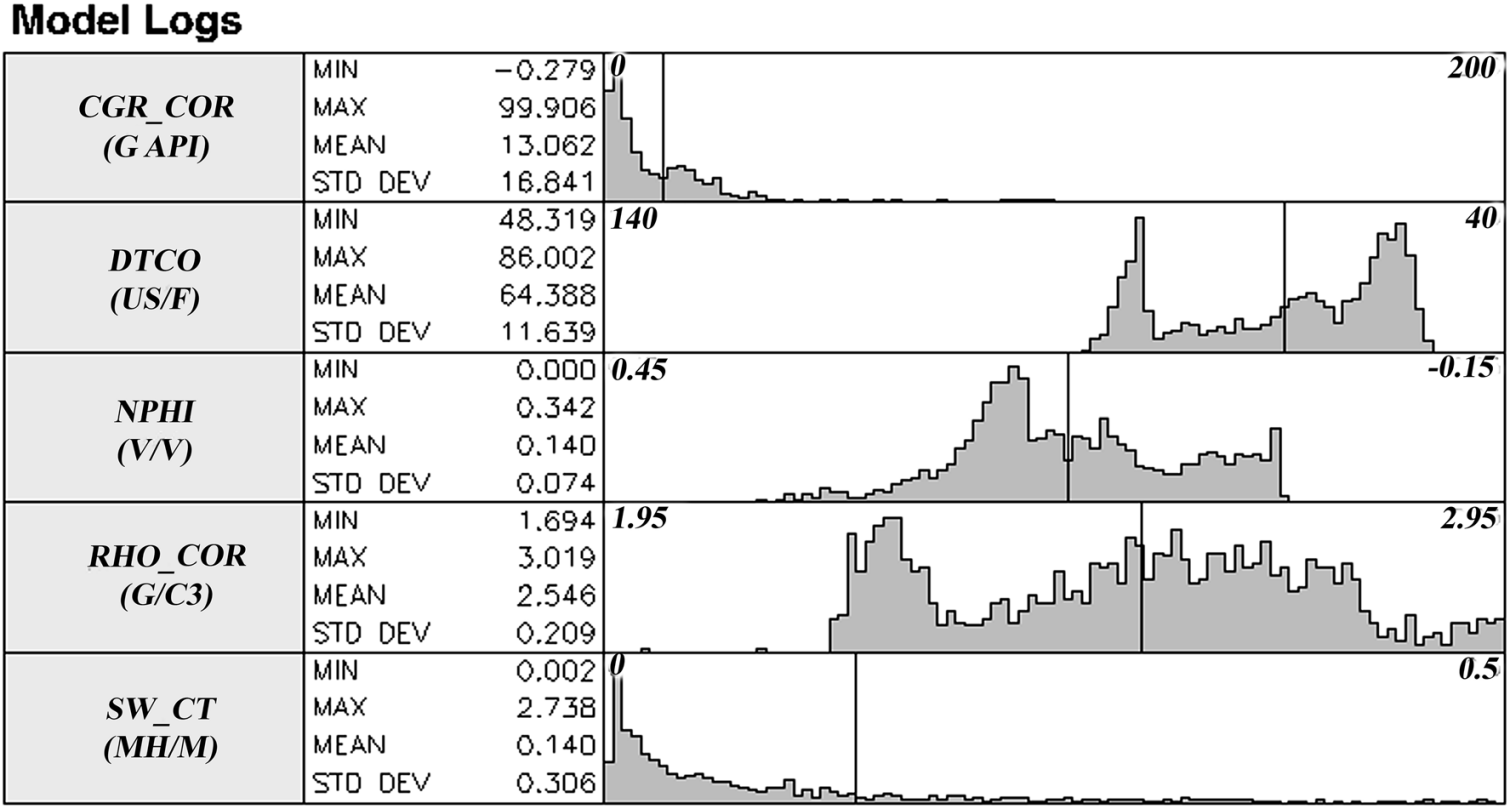
Frequency diagram of model input logs.

**Figure 12:**
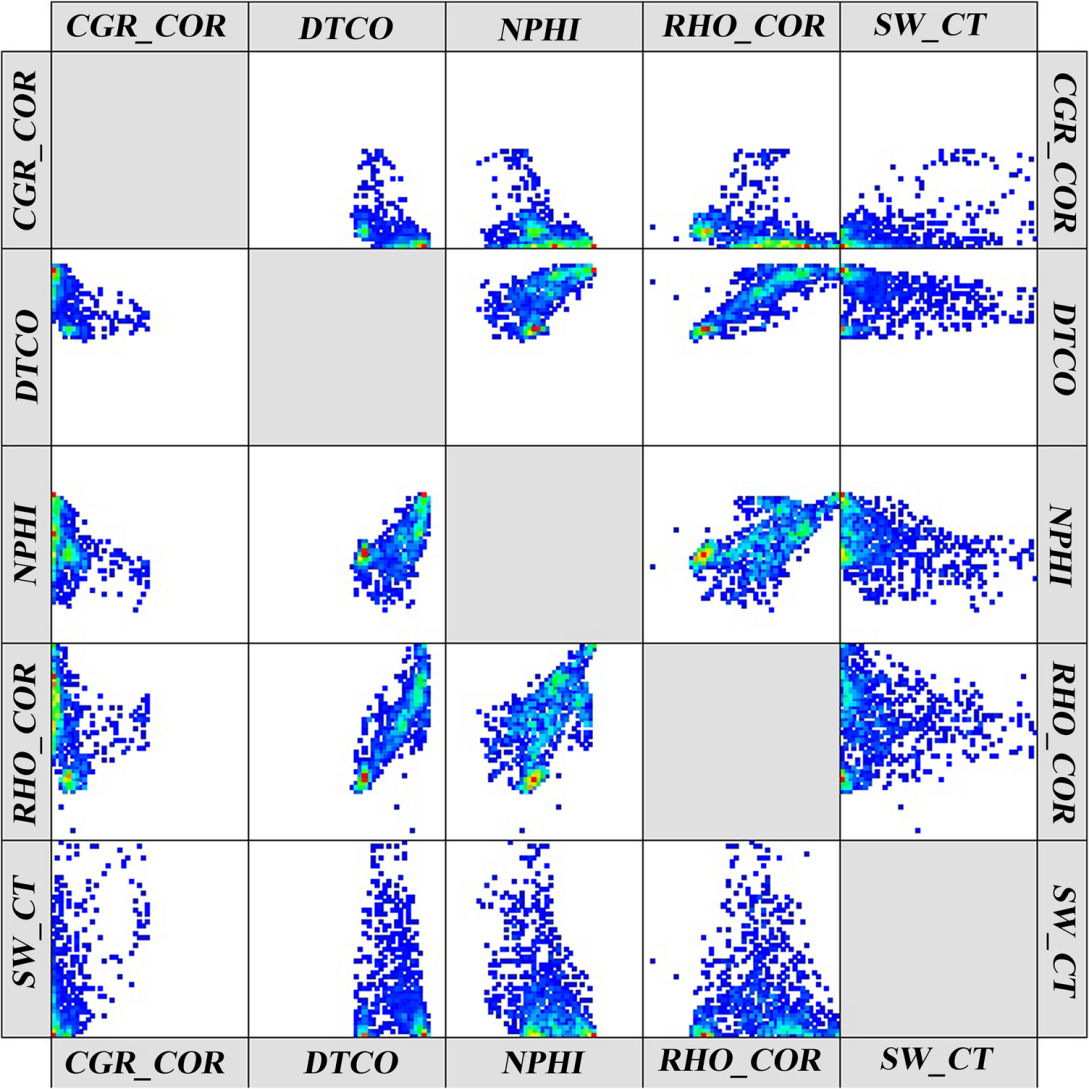
Cross-over diagram of model input logs relative to each other.

In this study, facies with common geological/reservoir properties are classified into different categories using readings of gamma, neutron, density, acoustic, and resistivity diagrams. Figures 11 and 12 show the frequency diagrams of the input logs of the model and cross diagrams of these logs, respectively.

## Discussion

### MRGC clustering description

Data clustering is the basis of modeling and classification algorithms. This process aims to define small natural and fundamental groups of large datasets^50,60^. The MRGC (Multi-resolution graph-based clustering) method is one of the few non-parametric and very suitable methods for studying and analyzing data clusters from the logs with the characteristics mentioned. In this method, the log data is indexed by two NI (Neighboring Index Parameter) that determine the position of the proximity of each point in a dataset to the summit or probability of the density function of the data and the KRI (Kernel Representative Index) is an indicator to determine the prone points to represent the core or center of the cluster.

**Figure 13:**
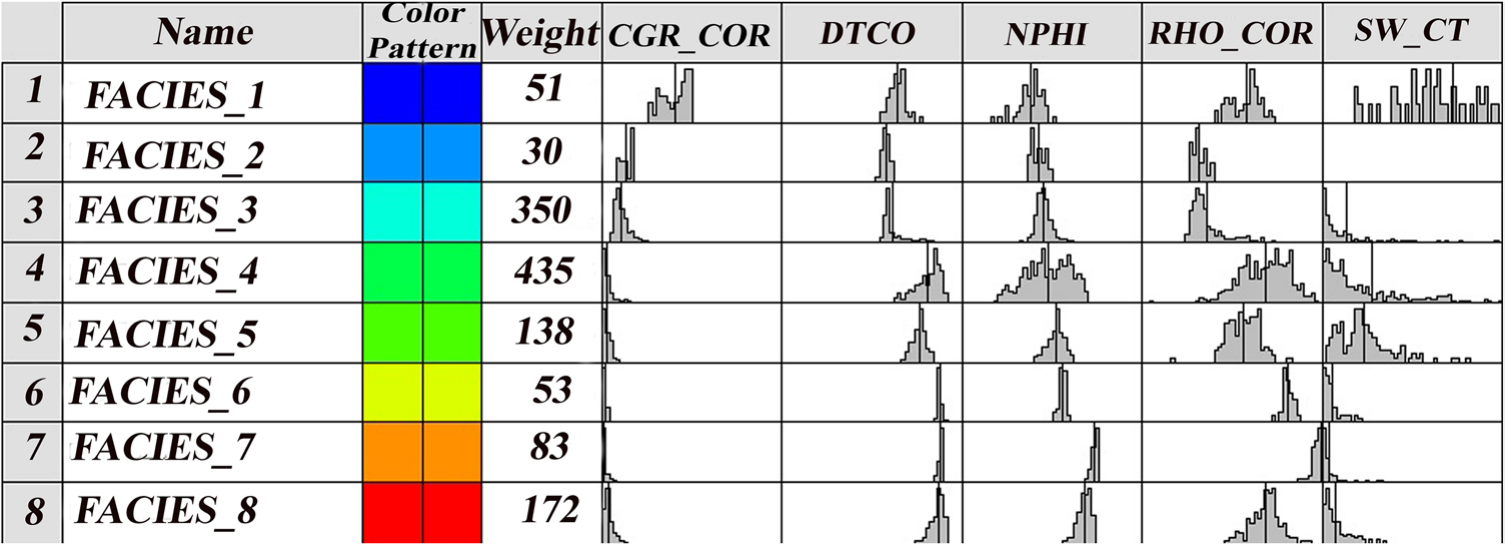
Facies produced by MRGC method.

In this study, based on the MRGC clustering method, the upper and lower limit of optimal data and models are determined. Finally, applying this model produced an optimal model with eight facies. The results of categorized facies are presented in Fig. 13. It has shown the readings of each model input log in the separated facies with their weight.

**Figure 14:**
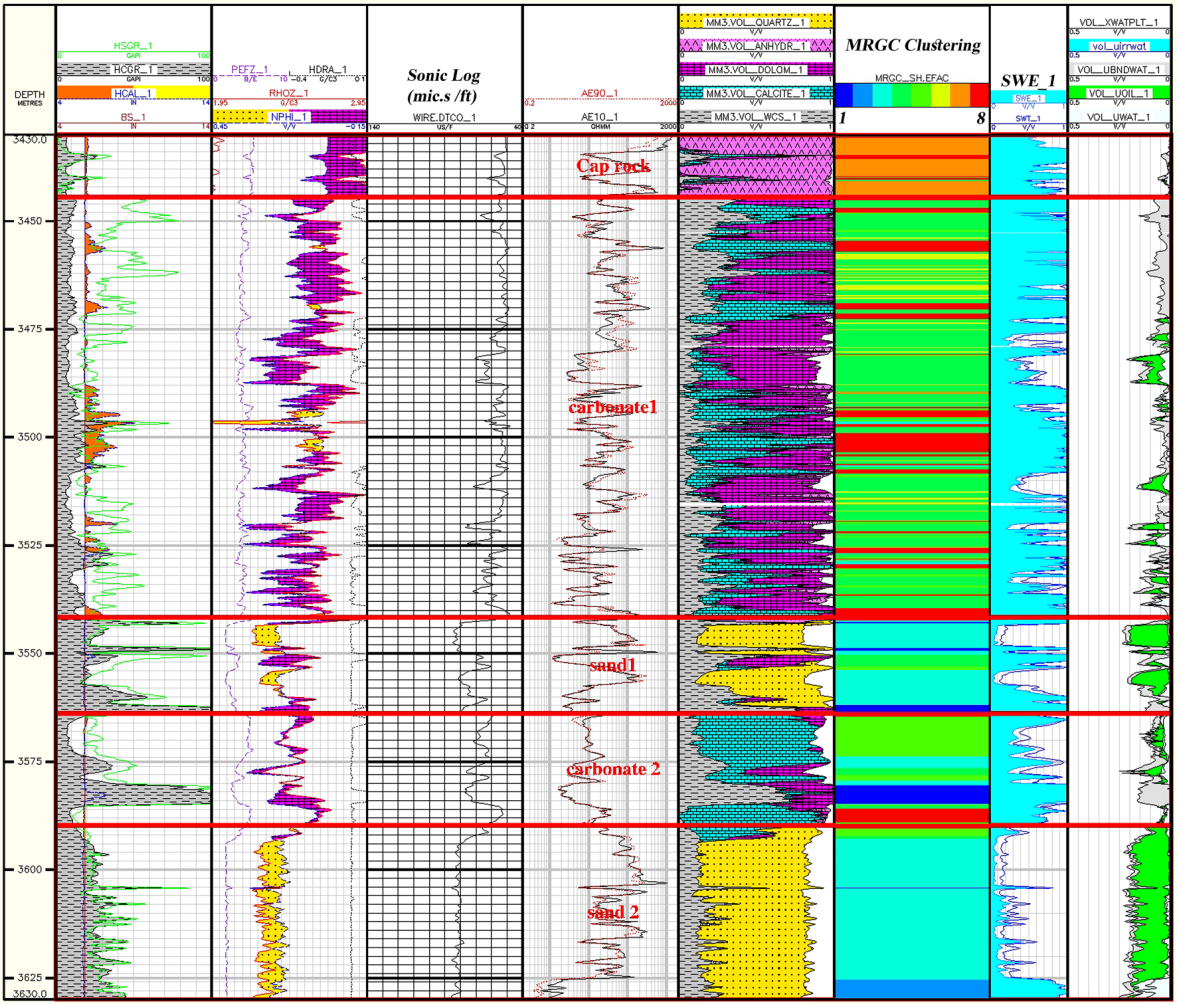
Classification of facies in the well sequence and final evaluation results using MRGC method.

Figure 14 shows the facies classification throughout the sequence of the Asmari Formation in the studied wells in the Mansouri Field. Furthermore, it has compared the lithofacies produced by the lithological column and the saturated and hydrocarbon columns. Comparing lithological columns in sandstone and carbonate lithologies and facies columns is clearly illustrated.

### ANN clustering description

**Figure 15:**
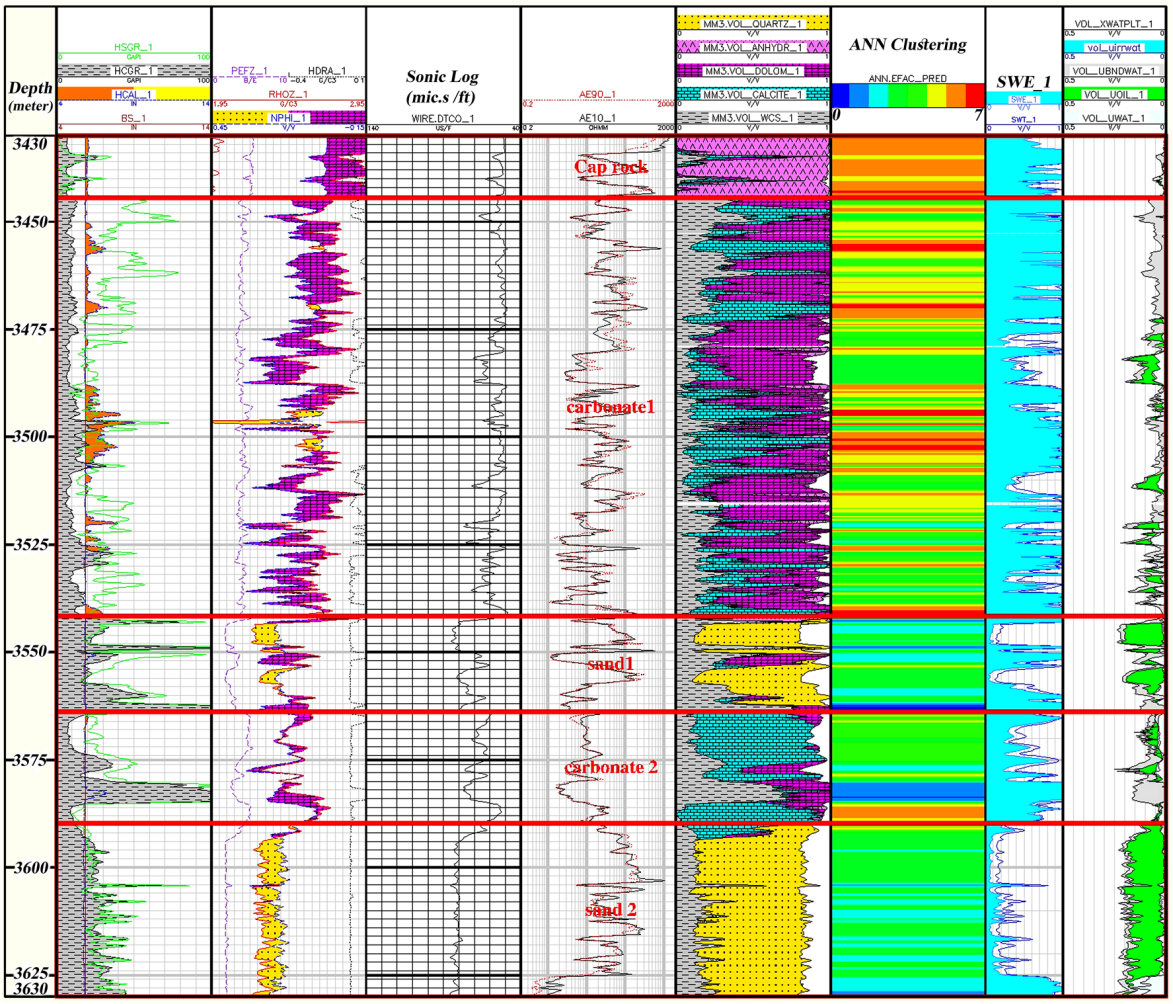
Classification of facies in the well sequence and final evaluation results using the ANN method.

An artificial neural network is a computerized model that attempts to emulate biological learning processes and simulate specific tasks of the human nervous system. Neurons are composed of neurons as microprocessors, each of which has a simple task. Based on the ANN clustering method, the present study assumed eight optimal facies in the previous stage to estimate an entire facies in the well by constructing an ANN model between petrophysical logs and the pre-facies logs. In constructing the neural network model, the Levenberg–Marquardt (L-M) algorithm trained the data. This network has three layers (input, hidden, and output). The number of neurons is calculated through trial and error and response optimization as 2-5-1. The facies classification throughout the sequence of the Asmari Formation is shown in Fig. 15.

**Figure 16:**
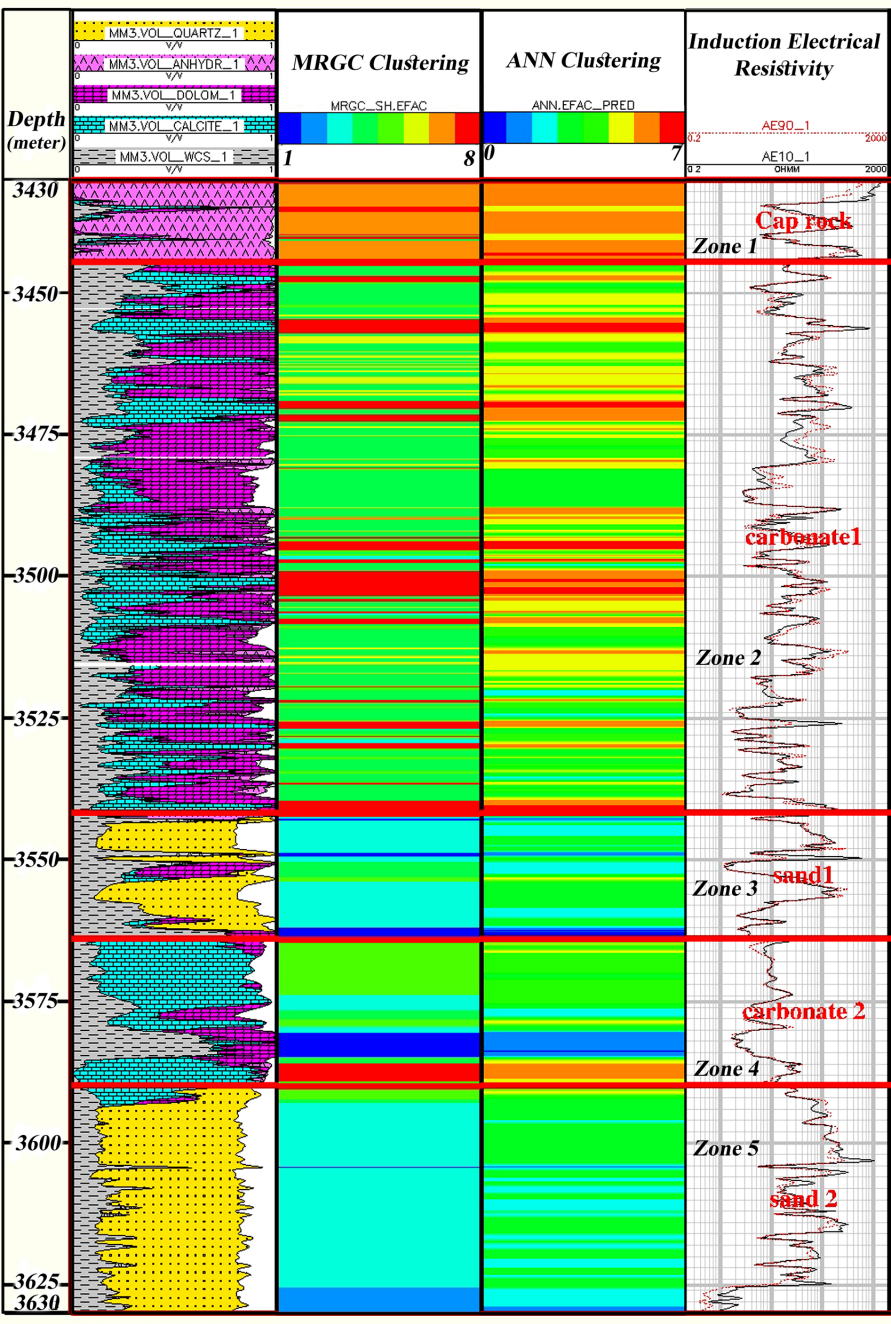
Comparing zoning results of the Geolog, MRGC, and ANN clustering techniques in the Asmari Formation.

Figure 16 shows the correlation and comparing zoning results of all three Geolog, MRGC, and ANN clustering techniques. However, the number of MRGC and ANN’s electrical facies classifications and lithofacies of the Geolog is different; similar results of correct separating of anhydrite, limestone, and sandstone are noticed, especially in zones three and five with dominant sandstone lithology.

### Geological lithofacies validating data

Based on previous microfacies studies, four sedimentary environments of open sea, dam, lagoon and coastal environment can be distinguished for the deposition of the Asmari Formation in this field, which is deposited on a platform with a low slope^18,24,29^. In this study, thin sections of the Asmari Formation in the studied well-A have identified 11 general facies. Asmari formation in the Mansouri field has clastic and carbonate components. Therefore, examining existing thin sections has led to the identification of two general facies: carbonate-evaporitic facies and siliceous-clastic petrofacies. In order to validate the results, the siliceous petrofacies data is used with the assumption that the electrofacies are not necessarily related to the lithofacies and that different facies can be placed inside a specific clustering zone. Siliceous-clastic petrofacies are describes as:

### Quartz arenite petrofacies

**Figure 17:**
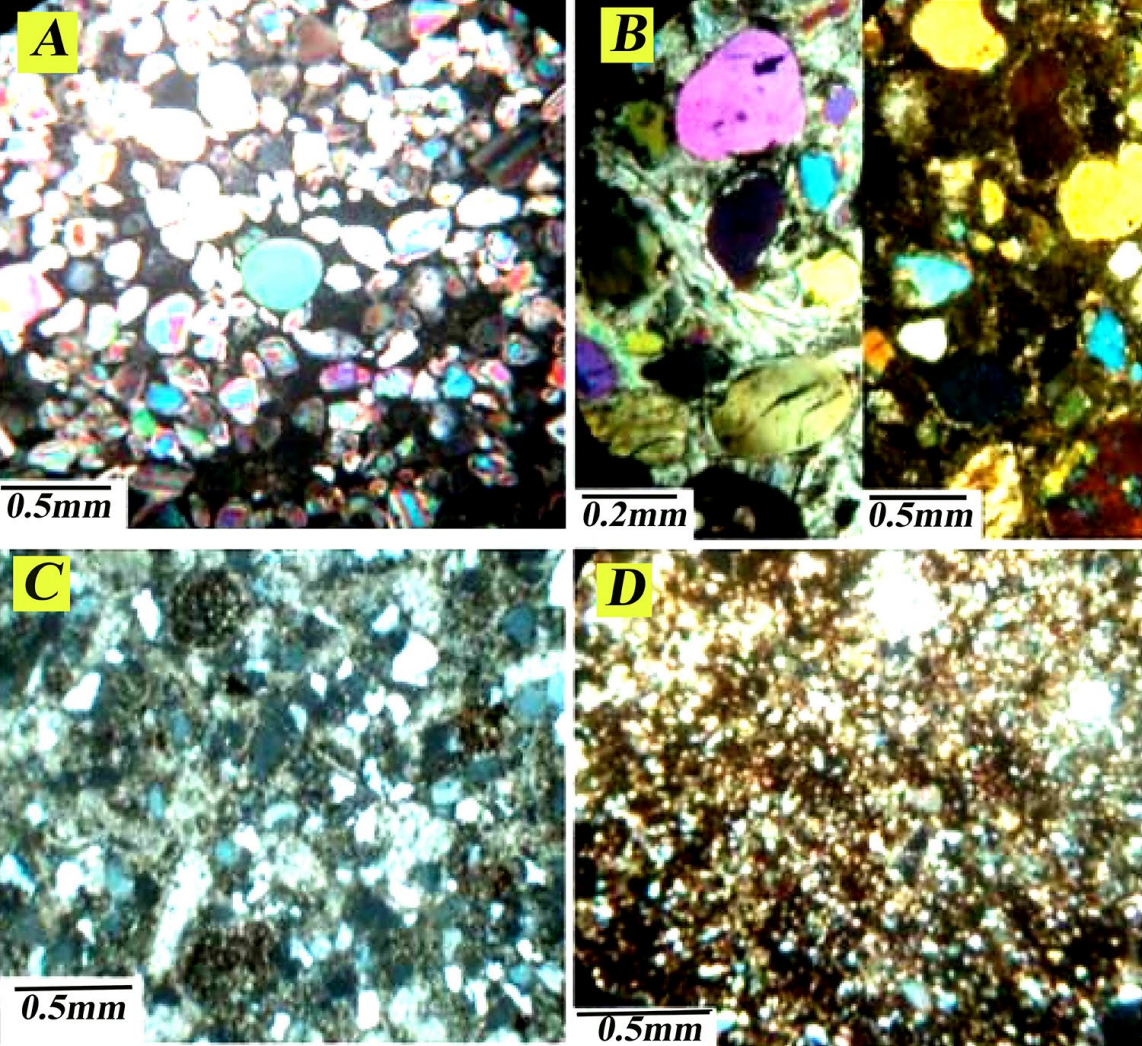
(**a**) Quartz arenite microfacies without cement, (**b**) quartz arenite microfacies with sulfate and dolomite cement, (**c**) sublitharenite petrofacies, (**d**) siltstone petrofacies of Well No. A, Mansouri oilfield.

This microfacies contains more than 95% quartz. Quartz particles are often angular and have suitable welding. In this field, sandstones are usually seen in two ways: (a) loose sand, where no cement can be seen between the quaternary particles. (b) Sandstones with carbonate or sulfate cement, carbonate cement often includes dolomite and sometimes micrite limestone, and sulfate cement also includes anhydrite. Due to the texture maturity and appropriate particle melting, these facies can be attributed to a coastal environment with high energy (Fig. 17a and b).

### Sublitharenite petrofacies

These facies comprise clastic and carbonate particles (skeletal grains) (Fig. 17c).

### Siltstone petrofacies

This petrofacies is usually deposited in low-energy environments. In the Asmari Formation, this facies is mainly seen in the lower parts, and its amount decreases towards the top of the Formation (Fig. 17d).

Based on the results of this study, different cavity systems with different petrophysical characteristics can be separated in the studied well in the Asmari Formation, and the facies with the best reservoir conditions can be determined.

**Figure 18:**
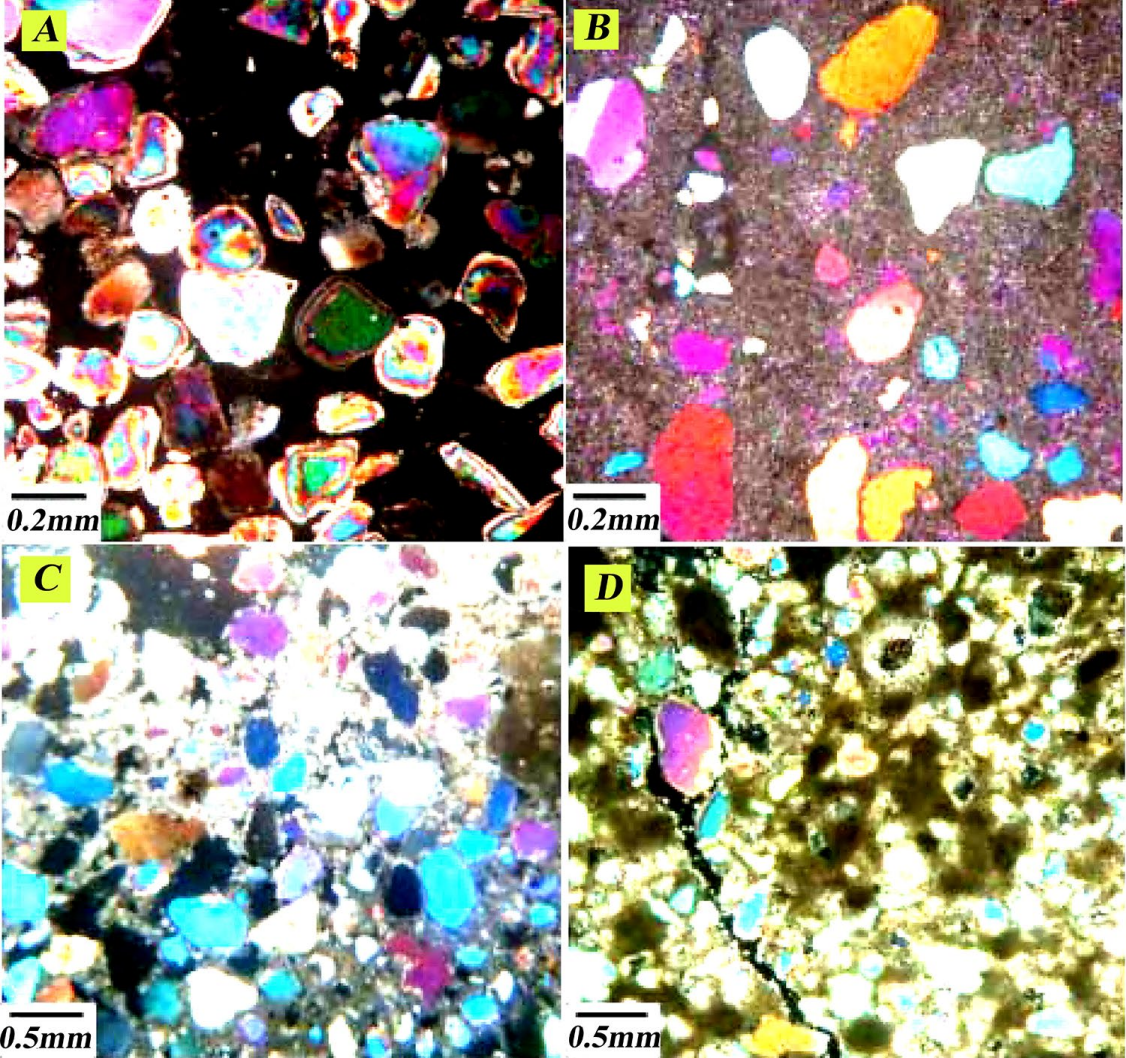
Drilling sample thin sections of the Asmari Formation in Mansouri field includes (**A**) without cement sandstorms due to migrating hydrocarbons before cementing, (**B**) dissolution in sandstones, (**C**) dolomite cement in sandstones, (**D**) dolomite dissolution in sandstones.

Among the cluster zoning, sandstone and shale in zones 3 and 5 shows high reservoir quality. On the other hand, according to the depth related to these zones, most of the facies that exist in these depths include sandstone and dolomite facies, and this is affected by the two factors of the primary sedimentary texture and the effect of the diagenesis process on them. Processes such as dissolution, migration of hydrocarbons to the reservoir before cementation of sandstones and dolomitization of cement have improved the quality of the reservoir in the Ahwaz sandstone section, which can be seen in the evaluated thin section (Fig. 18). As a result, the determined lithofacies are affected by diagenesis processes and the type of porosity created by these processes.

## Conclusions

Electrofacies are valuable approaches for recognizing and determining intervals with comparable petrophysical log responses and roughly equivalent lithologies within a formation almost homogeneous in composition and empty of biostratigraphic indicators or marker beds. Consequently, the confined lithofacies are influenced by diagenesis approaches and the process of porosity type. Based on a petrophysical study of 280 core samples from one of the one exploratory well drilled in the Asmari reservoir located in the Mansouri field, the following results are outlined:

- Determination of the production zones in the Asmari Formation Sequence is determined using the method. Accordingly, shear limits are considered based on the combination of different lithologies in designated zones for three parameters of effective porosity, effective water saturation, and shale volume. After applying these shear zones, the potential zones with high oil accumulation are identified.
- Petrophysical parameter facts depict the clear distinction between zones 3 and 5 in terms of the reservoir capacity of sandstone and shale.
- Regarding estimating electrical facies by MRGC and ANN methods, despite the differences in concepts and methods, these techniques are all exclusively powerful tools for predicting electrical facies.
- In addition, the results show that intelligent systems can successfully model patterns in test data when there is a logical relationship between input data or between input and output data. The model can be used for Recognition and making reasonable conclusions between actual and estimated data. Suppose the parameters necessary for making these models are adjusted, regardless of the lithology and formation characteristics in the relevant oilfield. In that case, these methods can also be applied to other field wells that lack specific facies.
- On the other hand, by comparing the performance of the parameters measured and estimated from different methods, it can be concluded that the MRGC method is more accurate and successful than other methods for determining these parameters in the wells.
- In conclusion, it is worth noting that MRGC is an efficient method for clustering and homogenizing data and determining different reservoir parameters, which, in addition to the high speed of operation, is not limited to data size and numbers.
- Comparing MRGC and ANN electrofacies and lithofacies demonstrate similar results of correct separating of anhydrite, limestone, and sandstone are noticed, especially in zones three and five with dominant sandstone lithology
- The siliceous petrofacies from describing thin sections are employed for data validation with the assumption that the electrofacies are not necessarily related to the lithofacies and that different facies can be placed inside a specific clustering zone. Accordingly, most of the facies that exist in zone 3 and 5 depths include sandstone and dolomite facies show comparable results with MRGC and ANN electrofacies.

It is suggested to considering core data from nearby wells in the Asmari Formation to more accurately evaluate and check the accuracy of rock type determination utilizing flow zone index (FZI) and fuzzy center mean (FCM). Furthermore, it is possible to use MRGC and ANN clustering zone to determine sandstone reservoir in nearby drilled wells and generalize the results to coreless wells in the Asmari Formation of Mansouri oilfield (Supplementary Information).

### Supplementary Information

Supplementary Information.

## Data Availability

The following datasets generated and/or analyzed during the current study are available in the Mahmoud Memariani repository, 10.13140/RG.2.2.19913.31847. The other datasets generated and/or analyzed during the current study are not publicly available due to not permitted to share by National Iranian Oil Company Exploration Directorate (NIOC-EXP) request but are available from the corresponding author on reasonable request.

## Abbreviations

ACE: Alternating conditional expectations
ANN: Artificial neural network
EF: Electrofacies
FZI: Flow zone index
HCA: Hierarchical cluster analysis
KPCA: Kernel principal component analysis
KRI: Kernel Representative Index
LF: Lithofacies
L-M: Levenberg–Marquardt algorithm
MRGC: Multi resolution graph based clustering
RKHS: Reproducing kernel Hilbert space
SONN: Self organized neural network
