# Supplementary material for: Electrical facies of the Asmari Formation in the Mansouri oilfield, an application of multi-resolution graph-based and artificial neural network clustering methods
Source: Sci Rep. 2024 Mar 2;14:5198. doi: 10.1038/s41598-024-55955-0 (PMC11319792; doi:10.1038/s41598-024-55955-0)
Supplement: Supplementary file 1 — Supplementary Information. [file 41598_2024_55955_MOESM1_ESM.pdf]

Supplementary and Appendix

Electrical facies of the Asmari Formation in the Mansouri oilfield, an application of multi-resolution graph-based and artificial neural network clustering methods

- The following datasets generated and/or analyzed during the current study are available in the **Mahmoud Memariani** repository, http://dx.doi.org/10.13140/RG.2.2.19913.31847.

- The other datasets generated and/or analyzed during the current study are not publicly available due to not permitted to share by National Iranian Oil Company Exploration Directorate (NIOC-EXP) request but are available from the corresponding author on reasonable request.

Appendices

Appendix A: Calculations per hydraulic flow unit.

**φ**	**K**	**φ_z**	**RQI**	**FZI**
0.063198	0.014	0.067462	0.014648	0.217128
0.064482	0.011	0.068926	0.012794	0.185614
0.064453	0.009	0.068893	0.011852	0.172042
0.051991	0.005	0.054842	0.009738	0.177558
0.077318	0.013	0.083797	0.012687	0.151402
0.050253	0.006	0.052912	0.011237	0.212371
0.056834	0.005	0.060259	0.009313	0.154558
0.095872	0.029	0.106038	0.01729	0.163056
0.086586	0.021	0.094794	0.015521	0.163734
0.044274	0.005	0.046325	0.010552	0.227787
0.079963	0.026	0.086912	0.018029	0.207443
0.08791	0.029	0.096383	0.01791	0.185822
0.054484	0.005	0.057623	0.009512	0.165077
0.065784	0.006	0.070417	0.009694	0.137667
0.071638	0.020	0.077166	0.01642	0.212788
0.052781	0.009	0.055722	0.012938	0.232184
0.05189	0.007	0.05473	0.011849	0.216507
0.053559	0.005	0.05659	0.009816	0.173463
0.05069	0.006	0.053397	0.010789	0.202049
0.075184	0.022	0.081296	0.017045	0.209661
0.068914	0.020	0.074015	0.016932	0.228771
0.052846	0.006	0.055794	0.01026	0.183893
0.04714	0.005	0.049472	0.009908	0.200278
0.067012	0.018	0.071825	0.016177	0.225228

Table A. 1 Classification of samples in hydraulic flow unit 1.

φ	K	φ_z	RQI	FZI
0.056197	0.019	0.059543	0.018051	0.303152
0.051977	0.018	0.054827	0.018424	0.336037
0.042978	0.011	0.044908	0.016051	0.357426
0.076202	0.047	0.082488	0.024713	0.299601
0.059959	0.025	0.063783	0.020415	0.320061
0.075312	0.040	0.081446	0.022996	0.282344
0.035064	0.005	0.036338	0.011857	0.326306
0.051394	0.011	0.054178	0.014699	0.271302
0.032845	0.006	0.033961	0.013404	0.394689
0.038309	0.005	0.039835	0.011344	0.284772
0.041934	0.005	0.04377	0.010843	0.247716
0.057777	0.020	0.06132	0.018631	0.303825
0.057514	0.015	0.061023	0.015989	0.262019
0.072351	0.033	0.077994	0.021163	0.271338
0.050514	0.021	0.053201	0.020317	0.381888
0.071272	0.034	0.076742	0.021581	0.281219
0.058492	0.025	0.062126	0.020452	0.329206
0.06485	0.021	0.069348	0.017757	0.256051
0.041184	0.012	0.042954	0.016757	0.390119
0.046734	0.006	0.049025	0.011561	0.235823
0.038004	0.005	0.039505	0.011389	0.288299
0.038582	0.005	0.040131	0.011593	0.288873
0.031336	0.005	0.032349	0.012543	0.387729
0.033449	0.005	0.034607	0.01214	0.350797
0.041145	0.007	0.042911	0.012499	0.291277
0.073945	0.048	0.07985	0.025366	0.317669
0.05916	0.022	0.06288	0.019129	0.304215
0.035466	0.005	0.03677	0.01179	0.320642
0.03528	0.005	0.03657	0.011821	0.323244
0.033824	0.005	0.035009	0.012073	0.344846
0.036395	0.006	0.03777	0.013032	0.345046
0.050225	0.010	0.052881	0.013987	0.264504
0.096394	0.096	0.106677	0.031329	0.293684
0.077551	0.041	0.08407	0.022922	0.272656
0.089585	0.055	0.0984	0.024702	0.251041
0.081767	0.041	0.089048	0.022169	0.248956
0.069363	0.050	0.074533	0.026554	0.356273
0.079032	0.064	0.085815	0.028153	0.328068
0.052569	0.013	0.055486	0.015854	0.285729
0.08457	0.050	0.092383	0.024263	0.262636
0.076449	0.044	0.082777	0.023891	0.288623
0.100821	0.091	0.112126	0.029899	0.266659
0.108246	0.107	0.121385	0.031236	0.25733
0.132739	0.272	0.153055	0.044959	0.293744
0.132408	0.220	0.152616	0.040454	0.265072
0.112474	0.102	0.126727	0.029854	0.235575
0.097852	0.085	0.108466	0.029189	0.269108
0.06115	0.015	0.065132	0.015766	0.24206
0.083736	0.053	0.091388	0.024871	0.272144
0.039232	0.005	0.040834	0.01121	0.27452
0.038628	0.005	0.04018	0.011297	0.281162
0.037366	0.005	0.038816	0.011486	0.295912
0.031245	0.005	0.032253	0.012561	0.38945
0.03542	0.007	0.03672	0.014049	0.382599
0.031272	0.005	0.032282	0.012555	0.388932
0.041403	0.005	0.043191	0.010912	0.252645

0.055882	0.018	0.059189	0.017866	0.301848
0.078684	0.045	0.085404	0.023722	0.277765
0.069682	0.027	0.074901	0.019567	0.261237
0.061573	0.031	0.065613	0.02235	0.340635
0.049709	0.018	0.052309	0.019037	0.363928
0.065471	0.023	0.070058	0.018439	0.263194
0.038365	0.009	0.039896	0.015259	0.382473
0.038093	0.007	0.039602	0.013446	0.339542
0.032797	0.005	0.033909	0.012454	0.367272
0.035464	0.005	0.036768	0.01179	0.320668
0.046595	0.010	0.048872	0.014531	0.297317
0.046366	0.015	0.04862	0.017801	0.366129
0.078307	0.063	0.08496	0.028131	0.331112
0.074928	0.069	0.080997	0.030239	0.373331
0.059494	0.024	0.063257	0.020087	0.317551
0.054871	0.020	0.058056	0.018789	0.323629
0.049744	0.015	0.052348	0.016962	0.324016
0.043935	0.010	0.045954	0.014787	0.321781
0.035401	0.005	0.0367	0.01226	0.334056
0.03699	0.007	0.038411	0.013181	0.343153
0.049413	0.019	0.051982	0.01926	0.370506
0.08865	0.097	0.097273	0.032896	0.338185
0.04981	0.010	0.052421	0.014113	0.269227
0.033556	0.005	0.034721	0.012121	0.349096
0.039186	0.008	0.040784	0.014114	0.346069
0.063419	0.022	0.067713	0.018407	0.271838
0.067868	0.054	0.072809	0.028055	0.385322
0.059935	0.034	0.063756	0.023565	0.369602
0.078203	0.062	0.084837	0.027987	0.329889
0.027967	0.003	0.028771	0.010207	0.354758
0.047577	0.014	0.049954	0.016878	0.337864
0.031774	0.004	0.032817	0.010536	0.321052
0.057159	0.013	0.060624	0.015047	0.248206
0.053551	0.017	0.056581	0.017723	0.313233
0.035713	0.006	0.037035	0.012915	0.348731
0.057259	0.031	0.060737	0.023058	0.379642
0.087786	0.101	0.096233	0.033691	0.350095
0.074246	0.070	0.0802	0.030504	0.380345
0.047594	0.011	0.049972	0.014948	0.299131
0.038918	0.005	0.040494	0.011255	0.277935
0.031735	0.005	0.032775	0.012024	0.366876
0.058631	0.019	0.062283	0.017735	0.284751
0.060052	0.033	0.063888	0.023247	0.363869
0.054752	0.014	0.057923	0.015609	0.26947
0.056063	0.023	0.059393	0.020211	0.340298
0.05807	0.031	0.06165	0.022872	0.370996
0.049793	0.011	0.052402	0.014644	0.279445
0.07891	0.037	0.08567	0.021484	0.250771
0.078355	0.056	0.085017	0.026641	0.313365
0.080129	0.064	0.087109	0.028073	0.322271
0.086451	0.084	0.094632	0.030943	0.326987
0.052178	0.021	0.05505	0.019699	0.357847
0.065362	0.040	0.069933	0.024617	0.352012

Table A. 2. Classification of samples in hydraulic flow unit 2.

φ	K	$φ_z$	RQI	FZI
0.078831	0.396	0.085577	0.070335	0.821896
0.116955	1.807	0.132445	0.123413	0.931807
0.028721	0.007	0.02957	0.015474	0.523308
0.052138	0.052	0.055006	0.03125	0.568126
0.065047	0.068	0.069573	0.03216	0.462246
0.030919	0.005	0.031906	0.012627	0.395756
0.022552	0.005	0.023072	0.014785	0.640829
0.02679	0.007	0.027527	0.016143	0.586446
0.03696	0.021	0.038378	0.023637	0.615904
0.029185	0.005	0.030063	0.012997	0.43232
0.034974	0.019	0.036241	0.023	0.634629
0.031089	0.008	0.032087	0.015491	0.482788
0.035041	0.011	0.036313	0.017655	0.48619
0.038041	0.018	0.039545	0.02149	0.54344
0.020891	0.005	0.021336	0.015362	0.719984
0.019741	0.005	0.020139	0.015803	0.784676
0.026498	0.008	0.027219	0.01692	0.621626
0.025873	0.005	0.026561	0.014182	0.533951
0.023244	0.005	0.023797	0.014563	0.611963
0.020224	0.005	0.020642	0.015613	0.756374
0.015992	0.005	0.016252	0.017557	1.080316
0.021351	0.005	0.021817	0.015195	0.696502
0.017611	0.005	0.017927	0.016731	0.93327
0.019751	0.005	0.020149	0.015799	0.784077
0.019433	0.005	0.019818	0.015927	0.803665
0.042937	0.044	0.044864	0.031879	0.710573
0.016201	0.005	0.016468	0.017444	1.059298
0.01759	0.005	0.017905	0.016741	0.935003
0.028908	0.005	0.029768	0.013059	0.438691
0.022599	0.006	0.023122	0.016675	0.721174
0.023084	0.005	0.023629	0.014614	0.618458
0.019545	0.005	0.019935	0.015882	0.796664
0.016306	0.005	0.016577	0.017387	1.048914
0.019014	0.005	0.019382	0.016102	0.830764
0.017666	0.005	0.017984	0.016705	0.928895
0.099565	1.130	0.110574	0.105766	0.956519
0.126208	0.670	0.144437	0.072332	0.500786
0.022786	0.005	0.023317	0.014709	0.63083
0.017637	0.005	0.017954	0.016719	0.931196
0.018987	0.005	0.019354	0.015703	0.81136
0.020421	0.005	0.020847	0.015143	0.726389
0.029289	0.005	0.030173	0.013341	0.442144
0.022697	0.005	0.023224	0.014717	0.633691
0.039898	0.015	0.041556	0.019175	0.461424
0.03527	0.016	0.03656	0.021278	0.581997
0.053532	0.031	0.05656	0.023742	0.419758
0.021672	0.011	0.022152	0.02224	1.00398
0.024522	0.005	0.025138	0.014179	0.564041
0.024328	0.005	0.024935	0.014235	0.570881
0.02184	0.008	0.022327	0.019098	0.855366
0.02951	0.005	0.030407	0.012925	0.42507
0.036461	0.010	0.03784	0.016719	0.44182
0.020196	0.009	0.020613	0.020751	1.006725
0.019175	0.004	0.01955	0.014993	0.766925
0.050656	0.031	0.053359	0.024609	0.461188
0.018382	0.005	0.018726	0.016376	0.874515
0.022438	0.006	0.022953	0.016741	0.729368
0.043146	0.018	0.045091	0.020286	0.44989
0.038578	0.010	0.040126	0.016093	0.401069
0.035084	0.010	0.036359	0.016627	0.457304
0.054743	0.032	0.057913	0.024027	0.414875

0.029575	0.011	0.030476	0.018855	0.618686
0.024135	0.007	0.024732	0.016473	0.666073
0.034132	0.012	0.035338	0.018439	0.521806
0.018638	0.008	0.018992	0.020186	1.062884
0.018089	0.005	0.018422	0.016508	0.896105
0.023346	0.005	0.023904	0.014531	0.6079
0.016959	0.005	0.017252	0.017323	1.0041
0.018709	0.007	0.019066	0.018799	0.986015
0.029309	0.023	0.030194	0.027856	0.922586
0.029843	0.013	0.030761	0.02072	0.673591
0.033778	0.009	0.034959	0.016135	0.461549
0.019377	0.005	0.01976	0.01595	0.807181
0.033258	0.011	0.034402	0.018388	0.534495
0.039319	0.022	0.040928	0.023308	0.569495
0.044574	0.023	0.046654	0.022627	0.484992
0.030319	0.005	0.031267	0.012751	0.407819
0.037558	0.010	0.039024	0.016378	0.419693
0.120264	0.447	0.136704	0.060568	0.443056
0.029119	0.020	0.029992	0.026199	0.87352
0.023117	0.005	0.023664	0.014603	0.617114
0.02446	0.005	0.025073	0.014197	0.566206
0.031483	0.014	0.032506	0.021047	0.647472
0.03675	0.016	0.038152	0.020598	0.539891
0.064123	0.047	0.068517	0.026934	0.393107
0.049096	0.041	0.051631	0.028588	0.553698
0.067165	0.059	0.072001	0.029433	0.408784
0.082343	0.138	0.089731	0.040658	0.453102
0.083778	0.207	0.091439	0.049349	0.539695
0.081508	0.146	0.088741	0.042088	0.474281
0.073107	0.107	0.078874	0.038069	0.48266
0.063387	0.045	0.067677	0.026541	0.392174
0.07622	0.105	0.082509	0.036886	0.44705
0.02023	0.004	0.020648	0.0141	0.682855
0.036782	0.012	0.038186	0.017698	0.463463
0.017219	0.005	0.017521	0.01692	0.965734
0.016219	0.005	0.016486	0.017434	1.057503
0.021238	0.005	0.021699	0.015236	0.70215
0.020583	0.005	0.021015	0.015973	0.760062
0.021028	0.005	0.02148	0.015311	0.712821
0.026057	0.007	0.026754	0.015895	0.594127
0.020819	0.005	0.021262	0.015388	0.723727
0.024957	0.017	0.025596	0.025553	0.998321
0.015676	0.005	0.015925	0.017734	1.113545
0.016025	0.005	0.016286	0.017539	1.076948
0.019547	0.005	0.019936	0.015881	0.796584
0.018733	0.005	0.019091	0.016222	0.849721
0.027997	0.005	0.028804	0.01327	0.460694
0.020137	0.005	0.020551	0.015647	0.761374
0.018032	0.007	0.018363	0.020188	1.099342
0.020667	0.005	0.021103	0.015611	0.739777
0.047102	0.030	0.04943	0.025189	0.509578
0.043826	0.015	0.045835	0.01819	0.396849
0.043293	0.028	0.045252	0.025416	0.561648
0.052512	0.031	0.055423	0.024316	0.438746
0.030054	0.005	0.030985	0.012397	0.400105
0.034468	0.009	0.035699	0.015628	0.437781

Table A. 3. Classification of samples in hydraulic flow unit 3.

φ	K	$φ_z$	RQI	FZI
0.053569	0.254	0.056601	0.068399	1.20843
0.016088	0.012	0.016351	0.027211	1.664173
0.034436	0.082	0.035665	0.048328	1.355081
0.02706	0.034	0.027813	0.035434	1.27402
0.043741	0.238	0.045742	0.07318	1.599828
0.012539	0.005	0.012698	0.019828	1.561474
0.014241	0.005	0.014447	0.018606	1.287851
0.013211	0.005	0.013387	0.019318	1.442964
0.014663	0.005	0.014881	0.018336	1.23215
0.032394	0.102	0.033478	0.055786	1.666327
0.015387	0.005	0.015628	0.017899	1.145359
0.014466	0.005	0.014678	0.01846	1.25768
0.015489	0.005	0.015733	0.01784	1.133955
0.011627	0.005	0.011764	0.020051	1.704415
0.023185	0.020	0.023735	0.029196	1.230088
0.028175	0.037	0.028992	0.035818	1.235458
0.017229	0.011	0.017531	0.025044	1.428609
0.013854	0.005	0.014048	0.018864	1.342811
0.011244	0.005	0.011372	0.020939	1.841193
0.012201	0.005	0.012352	0.021053	1.704486
0.012206	0.005	0.012357	0.020097	1.626425
0.01722	0.015	0.017521	0.029701	1.695125
0.020192	0.020	0.020608	0.030924	1.500615
0.015612	0.005	0.01586	0.01777	1.1204
0.011563	0.005	0.011699	0.020648	1.764982
0.016811	0.018	0.017098	0.03218	1.88211
0.012923	0.005	0.013092	0.019532	1.49191
0.013885	0.005	0.01408	0.018843	1.338218
0.011894	0.005	0.012038	0.020358	1.691242
0.024328	0.032	0.024935	0.036265	1.4544

Table A. 4. Classification of samples in hydraulic flow unit 4.
